# Amyotrophic Lateral Sclerosis and Frontotemporal Dementia Have Distinct Prediagnostic Blood Biochemical Profiles

**DOI:** 10.1002/ana.78082

**Published:** 2025-10-30

**Authors:** Christos V. Chalitsios, Jiali Gao, Carol A.C. Coupland, Julia Hippisley Cox, Martin R. Turner, Alexander G. Thompson

**Affiliations:** ^1^ Nuffield Department of Clinical Neurosciences University of Oxford Oxford UK; ^2^ School of Medicine University of Nottingham Nottingham UK; ^3^ Wolfson Institute of Population Health Queen Mary University London UK

## Abstract

**Objective:**

Identifying modifiable factors influencing amyotrophic lateral sclerosis (ALS) and frontotemporal dementia (FTD) risk is important for prevention. Blood biomarkers, particularly cholesterol, have been associated with neurodegenerative risk, but findings in ALS are inconsistent, and data on FTD are limited.

**Methods:**

We conducted a population‐based cohort study using UK primary care records from QResearch linked with Hospital Episode Statistics. Adults with biomarker data recorded between 1998 and 2023 were included. We examined associations of low‐ and high‐density lipoprotein cholesterol (LDL‐C and HDL‐C), total cholesterol, triglycerides, creatinine, creatine kinase, and HbA1c with ALS and FTD risk. Cox proportional hazards models were used to estimate associations. Two‐sample Mendelian randomization (MR) was applied to explore genetically predicted associations of selected biomarkers.

**Results:**

There were up to 2,695 ALS and 781 FTD diagnoses, with a median follow‐up duration of 9.4 and 10.5 years, respectively. Higher LDL‐C (hazard ratio [HR]_per 1‐SD_ = 1.07, 95% confidence interval [CI] = 1.02–1.11) and total cholesterol levels (HR_per 1‐SD_ = 1.06, 95% CI = 1.02–1.10) were linearly associated with higher ALS risk. Age‐stratified analysis showed a stronger association for total cholesterol in those ≥ 60 years (HR_per 1‐SD_ = 1.08, 95% CI = 1.04–1.13, P_interaction_ = 0.003). Higher creatinine was inversely associated with FTD risk (HR_per 1‐SD_ = 0.90, 95% CI = 0.83–0.97), supported by MR (odds ratio [OR] inverse variance weighted (_IVW_)_, per 1‐SD_ = 0.73, 95% CI = 0.56–0.96). HbA1c showed a U‐shaped association with FTD (P_non‐linearity_ = 0.006).

**Interpretation:**

LDL and total cholesterol may provide insights into early disease changes or the etiology of ALS, whereas creatinine and HbA1c may be relevant for FTD. Research in monogenic ALS and FTD is needed to determine whether these biomarkers inform targeted prevention or intervention strategies. ANN NEUROL 2026;99:844–856

Amyotrophic lateral sclerosis (ALS) is a neurodegenerative disorder affecting the corticomotoneuronal system, leading to progressive motor neuron loss, muscle weakness, and death from neuromuscular respiratory failure, typically within 3 years of symptom onset.^1^ Frontotemporal dementia (FTD) is characterized by cognitive, behavioral, or language dysfunction at onset, progressing to more widespread impairments, and eventually death due to the preferential loss of neurons in the frontal and temporal lobes.^2^ ALS and FTD overlap in clinical, genetic, and pathological features. Up to 15% of patients with ALS meet the criteria for FTD with nearly 50% exhibiting cognitive or behavioral dysfunction on formal testing.^3^ The presence of insoluble neuronal and glial cytoplasmic inclusions of the ubiquitinated protein TDP‐43 is a pathological hallmark in 97% of ALS and 50% of FTD cases.^4^

Several genetic variants are associated with both ALS and FTD, with the most common being an intronic hexanucleotide repeat expansion (HRE) in *C9orf72*, inherited in an autosomal dominant manner.^5^ The *C9orf72* HRE can lead to ALS, FTD, or both within the same family.^6^ Additionally, rare variants in other genes have been linked to both conditions, with a monogenetic cause identified in approximately 11% of ALS and 18% of FTD cases.^7^, ^8^, ^9^ As genetic testing in ALS and FTD becomes widespread, an increasing number of people – first degree relatives of those with genetic ALS or FTD – become aware of their heightened risk. Identifying potentially modifiable factors that influence risk is therefore increasingly important, both for potential prevention strategies and for improving early detection in at‐risk individuals.

Epidemiological studies have begun to define a range of pre‐diagnostic metabolic changes in ALS, incorporating alterations in lipid, glucose, and energy metabolism, body mass index (BMI), and physical activity, and alterations in muscle markers.^10^, ^11^, ^12^, ^13^, ^14^ In particular, both observational and genetic epidemiological studies have implicated higher low‐density lipoprotein cholesterol (LDL‐C) levels as a potential causal risk factor for ALS.^15^ However, the role of lipid biomarkers in FTD remains unexplored in observational settings. Some studies have suggested that altered glucose metabolism may precede FTD diagnosis, and Mendelian randomization (MR) has implicated higher apolipoprotein B levels—the primary apolipoprotein of LDL‐C—as increasing FTD risk.^15^

Despite these findings, existing studies vary in methodology, are limited by small sample sizes, and lack direct comparisons between metabolic biomarkers and across ALS and FTD risk. This study, therefore, aims to systematically investigate the association of seven blood biomarkers—LDL‐C, high‐density lipoprotein cholesterol (HDL‐C), total cholesterol, triglycerides, creatinine, creatine kinase, and hemoglobin A1c (HbA1c)—with the risk of developing ALS or FTD in a large‐scale, real‐world primary care dataset from the United Kingdom.

## Methods

### Ethical Approval

This study was approved by the QResearch Scientific Committee on March 4, 2024 (Research ethics reference: 23/EM/0166, project reference: OX166). A dedicated webpage for this project is available on the QResearch website https://www.qresearch.org/research/research‐programs‐and‐projects/defining‐the‐relationship‐between‐lipids‐statins‐and‐the‐risk‐of‐amyotrophic‐lateral‐sclerosis‐and‐frontotemporal‐dementia‐using‐primary‐health‐records/. The lay summary for this study, research protocol, and statistical analysis plan are available from this webpage. QResearch is a Research Ethics Approved Database, confirmed by the East Midlands – Derby Research Ethics Committee.

### Study Design

**FIGURE 1:**
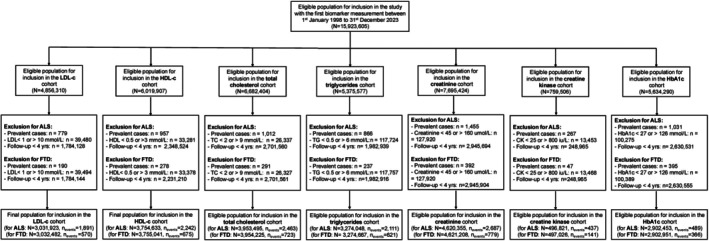
Flowchart depicting the selection process of study participants across different biomarker cohorts to analyze ALS and FTD outcomes. The initial eligible population includes participants with their first biomarker measurement recorded between January 1, 1998, and December 31, 2023. Each cohort was defined based on a specific biomarker (LDL‐C, HDL‐C, total cholesterol, triglycerides, creatinine, creatine kinase, and HbA1c), with exclusions applied for prevalent ALS or FTD cases, biomarker‐specific thresholds, and less than 4 years of follow‐up. The final population for inclusion in each cohort is presented separately for ALS and FTD outcomes. ALS = amyotrophic lateral sclerosis; CK = creatine kinase; FTD = frontotemporal dementia; HbA1c = hemoglobin A1c; LDL‐C = low‐density lipoprotein cholesterol; HDL‐C = high‐density lipoprotein cholesterol; TC = total cholesterol; TG = triglycerides.

To investigate the association among 7 blood biomarkers – total cholesterol, LDL‐C, HDL‐C, creatinine, triglycerides, creatine kinase, and HbA1c – and the risk of ALS and FTD, we conducted a population‐based cohort study using the QResearch database (version 47). Seven overlapping cohorts were constructed, each focusing on one biomarker, with ALS and FTD as the 2 outcomes of interest (Fig 1).

To complement our observational findings, we performed a 2‐sample MR analysis (see Supplementary Methods) to evaluate the genetic association of creatinine and HbA1c with ALS and FTD. MR analyses for lipid biomarkers were not repeated, as this has been published previously.^15^

### Data Sources

QResearch is a large electronic medical records database including primary care data from people registered across 1,423 general practices (18% of the English population) linked to hospital episode statistics (HES) and mortality register data (Office for National Statistics [ONS], NHS England). QResearch is based on anonymized medical records and data collected during clinical care and recorded on the Egton Medical Information Systems (EMIS) commercial computer system.

### Study Population

The study population for each biomarker cohort included individuals with their first biomarker measurement within the study period (January 1, 1998 to December 31, 2023). Participants were between 18 and 100 years old at the time of their first biomarker measurement (defined as the index date) and had been registered with a practice for at least 12 months before that measurement to ensure adequate recording of covariates. We excluded extreme biomarker values below the first and above the 99th percentile. Specifically, participants with HDL‐C < 0.5 or > 3 mmol/L, LDL‐C < 1 or > 10 mmol/L, total cholesterol < 2 or > 9 mmol/L, triglycerides < 0.5 or > 6 mmol/L, creatinine < 45 or > 160 umol/L, creatine kinase < 25 or > 800 iu/L, and HbA1c < 27 or > 126 mmol/L were excluded to reduce skew by recording errors or acutely deranged results.

### Definition of Outcomes

The outcomes were incident ALS and FTD diagnoses (separately). To reduce the likelihood of reverse causality (given that diagnostic latency in ALS and FTD is usually greater than 1 year from symptom onset and preceded by a presymptomatic phase of neurodegeneration^16^), those diagnosed with ALS or FTD within 4 years of the first biomarker measurement or with less than 4 years follow‐up after the first biomarker measurement and prevalent cases were excluded. ALS and FTD diagnoses were defined as the first recorded diagnosis of ALS or FTD in general practitioner or HES records during follow‐up (Supplementary Table S1).

### Covariates

We considered the following variables as potential confounding factors: age, sex, practice, Townsend deprivation index (a measure of material deprivation including four domains [unemployment, non‐car ownership, non‐home ownership, and household overcrowding]),^17^ smoking, body mass index (BMI), comorbidities (atrial fibrillation, cardiovascular diseases, peripheral vascular diseases, diabetes, and chronic kidney disease), prescriptions of anti‐hypertensive, anti‐diabetic medication, and lipid‐lowering therapy (LLT; full lists are provided in the Supplementary Materials). Smoking and BMI were assigned using data recorded at the time closest to the index date. Each comorbidity was defined as present if a relevant diagnostic code of that comorbidity was recorded at least once before the patient's index date.

### Statistical Analysis

Descriptive statistics were used to characterize the demographic and clinical features of the study cohort. Cox proportional hazard models were used to estimate the effect size with 95% confidence interval (CI) for the association of biomarkers with ALS and FTD incidence with age during follow‐up as the time scale. Robust standard errors were adopted to allow for intragroup correlation related to possible clustering effects by practice. The Cox proportional hazards assumption was checked by visual inspection of log(−log) plots and was largely satisfied. Follow‐up started from the first biomarker measurement until the occurrence of the outcomes of interest (ALS and FTD) or censoring, whichever occurred earlier. Censoring included death, transfer‐out from the practice, or the end of the study (December 31, 2023).

Models were constructed considering the first measurement of each biomarker as continuous (per 1 SD) and categorical variables (tertiles). For a better approximation of a normal distribution, a logarithmic transformation was applied to triglycerides, creatine kinase, and HbA1c levels when modeled as continuous variables. Models were adjusted for sex (male or female) and Townsend deprivation index (quintiles) and, as age was used as the underlying time scale, the effect of age at follow‐up on the risk of ALS and FTD was effectively considered.^18^ Additional adjustments were made specific to each cohort: for the lipid biomarker cohorts, we included LLT use as a time‐varying covariate (ie, patients were first considered non‐users and then users after their first prescribed/dispensed LLT); for the HbA1c cohort, we adjusted for a history of diabetes and anti‐diabetic medication use as a time‐varying covariate (ie, patients were first considered non‐users and then users after their first prescribed/dispensed anti‐diabetic medication); and for the creatinine cohort, we accounted for a history of chronic kidney disease. A stratified analysis by median age at sampling, sex, and LLT was also conducted to examine potential effect modification by these factors. Any subgroup differences were examined by testing the interaction term between the stratifying variable and each biomarker. As a secondary analysis, a Cox proportional hazard model with restricted cubic splines was also used to examine for any nonlinear association. Knots were placed at the 5th, 50th, and 95th percentiles, with the 50th (median) as the reference.

Finally, several sensitivity analyses were performed, repeating the analyses (a) excluding those with a history of FTD and ALS from the ALS and FTD cohorts, respectively, (b) including in the LDL‐C cohort, people with LDL‐C greater than 10 mmol/L (up to 69 mmol/L), as this might indicate familial hypercholesterolemia instead of a probable recording error, (c) after imputing missing values for BMI and smoking using multiple imputations by chained equations (MICE)^19^ with 5 imputed datasets and 5 iterations using the random forest method, a fully adjusted model was created by adding smoking status (non‐smoker, ex‐smoker, or current smoker), BMI (continuous), comorbidities (cardiovascular diseases, peripheral vascular diseases, and atrial fibrillation), and prescriptions for anti‐hypertensive medication as covariates.

To account for multiple comparisons, we applied false discovery rate (FDR) correction using the Benjamini‐Hochberg procedure in the primary analysis. Uncorrected *p* values of less than 0.05 were considered statistically significant for the subgroup analyses due to lower statistical power. Analyses were performed using Stata version 18 and R software version 4.3.1 (R Foundation for Statistical Computing, Vienna, Austria).

## Results

### LDL, HDL, Total Cholesterol, and Triglycerides

#### Cohort Characteristics

Full details of the demographic characteristics of the 4 cohorts used in the analysis of lipid biomarkers are presented in Supplementary Tables S2 to S5. The total number of participants with suitable data for analysis ranged from 3.03 to 3.95 million. During the follow‐up period, there were 1,898 to 2,471 ALS diagnoses and 573 to 726 FTD diagnoses, with a median follow‐up duration of 8.8 to 10.3 years. The median age at sampling ranged from 62.7 to 63.8 years for ALS and FTD, respectively.

#### Association With Amyotrophic Lateral Sclerosis and Frontotemporal Dementia

**FIGURE 2:**
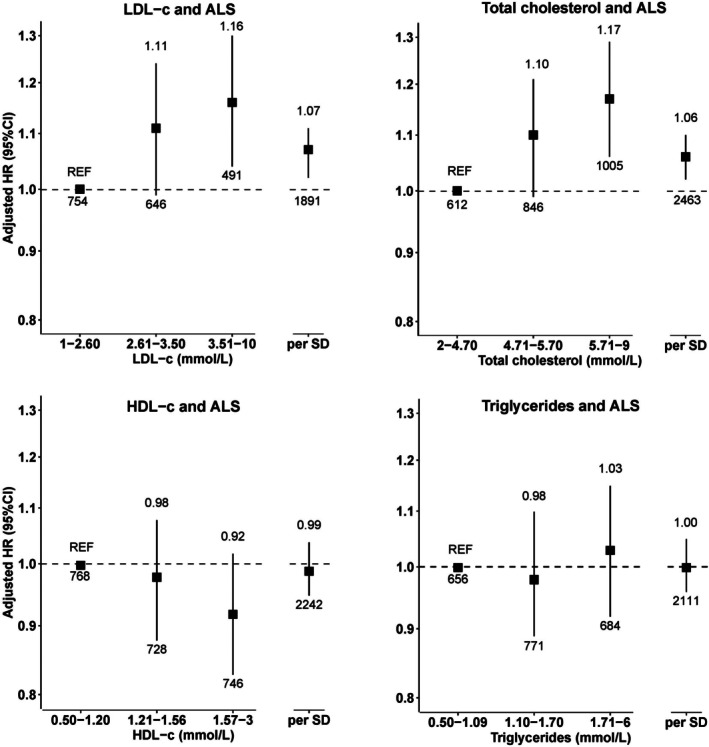
Association of the first measurement of 4 lipid blood biomarkers with the incidence of ALS. The numbers at the end of the lower and upper CI indicate, the total number of events and the effect estimate, respectively. ALS = amyotrophic lateral sclerosis; CI = confidence interval; HDL‐C = high‐density lipoprotein cholesterol; LDL‐C = low‐density lipoprotein cholesterol.

Higher levels of LDL‐C were linearly associated with an increased ALS risk (hazard ratio [HR]_per 1‐SD_ = 1.07, 95% CI = 1.02–1.11, n_events_ = 1,891; Fig 2, and Supplementary Table S6). Furthermore, compared with people in the lowest tertile of LDL‐C levels (1.00–2.60 mmol/L), those in the highest tertile (3.51–10.00 mmol/L) had a significantly higher risk of ALS (HR = 1.16, 95% CI = 1.04–1.20), suggesting a dose–response association (P_trend_ = 0.008). Similarly, total cholesterol levels were linearly associated with ALS risk, with a 6% increase in risk (HR_per 1‐SD increase_ = 1.06, 95% CI = 1.02–1.10, n_events_ = 2,463). People in the highest tertile of total cholesterol levels (5.71–9 mmol/L) had a 17% higher risk of ALS compared with those in the lowest tertile (2–4.70 mmol/L, HR = 1.17, 95% CI = 1.06–1.29, P_trend_ = 0.002). These associations remained statistically significant after FDR correction.

There was limited evidence of differences by sex and LLT use in the associations between lipid biomarkers and the risk of ALS (Supplementary Tables S7). However, when stratified by median age, higher total cholesterol was associated with a higher risk of ALS in people aged 60 years and older (HR_per 1‐SD_ = 1.08, 95% CI = 1.04–1.13) but not in those under 60 years (HR_per 1‐SD_ = 0.96, 95% CI = 0.88–1.05, P_interaction_ = 0.003; see Supplementary Table S7).

**FIGURE 3:**
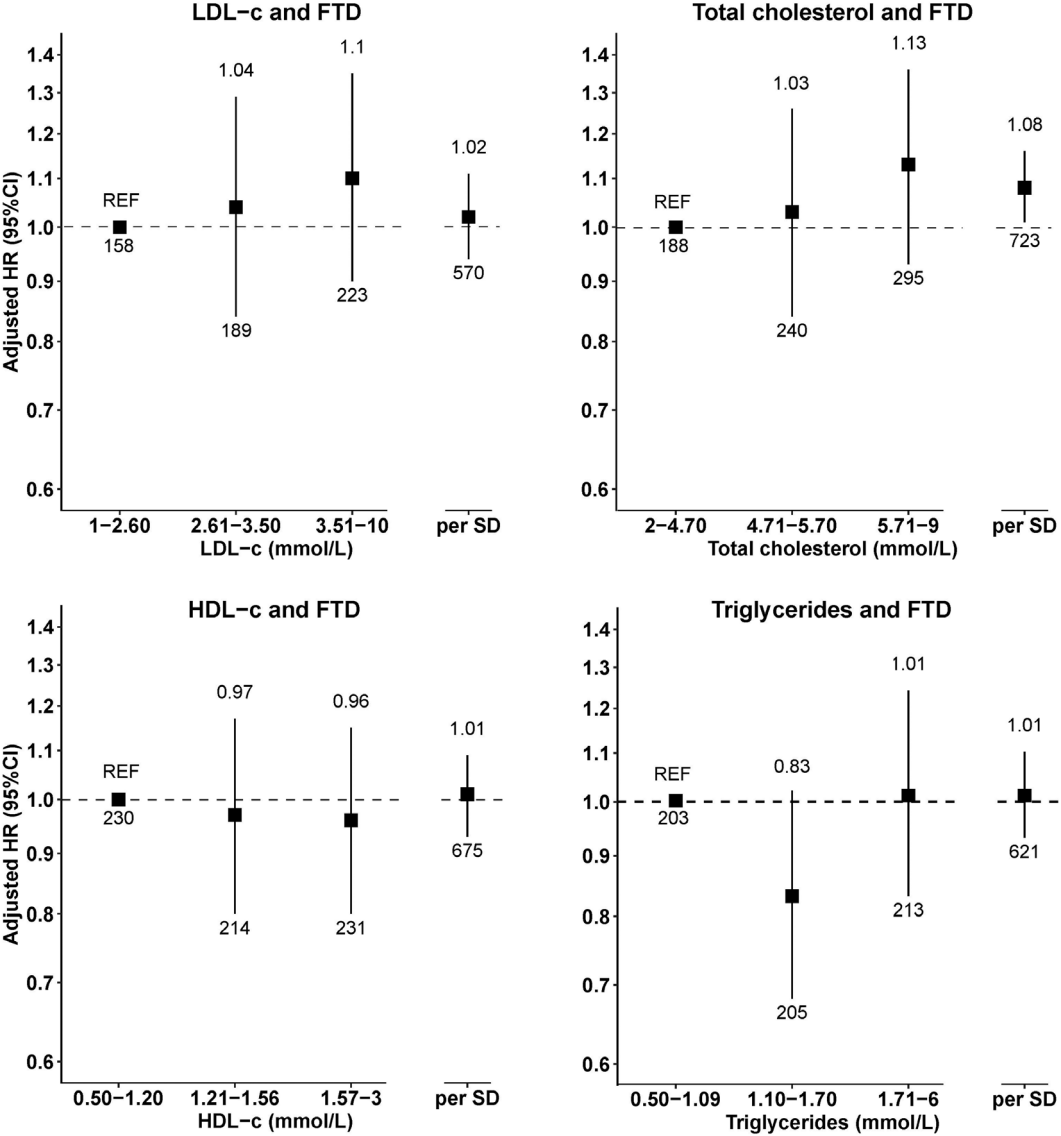
Association of the first measurement of 4 lipid blood biomarkers with the incidence of FTD. The numbers at the end of the lower and upper CI indicate, the total number of events and the effect estimate, respectively. CI = confidence interval; FTD = frontotemporal dementia; HDL‐C = high‐density lipoprotein cholesterol; LDL‐C = low‐density lipoprotein cholesterol.

No significant associations were observed among HDL‐C, LDL‐C, or triglyceride levels and the risk of FTD after FDR correction, although total cholesterol was nominally associated with an increased risk of FTD (HR_per 1‐SD increase_ = 1.08, 95% CI = 1.01–1.16, n_events_ = 723; Fig 3 and Supplementary Table S8). This was not supported by evidence of a dose–response association (P_trend_ = 0.192). Subgroup analyses indicated no effect modification by age, sex, and LLT use (Supplementary Table S9).

The associations among lipid traits, ALS, and FTD were also assessed using restricted cubic splines, which did not indicate evidence of nonlinearity (Supplementary Fig S1). The sensitivity analyses did not alter the above findings, except for attenuating the association between total cholesterol and FTD when additional covariates were included in the model (Supplementary Tables S10–S14).

### Creatine Kinase, Creatinine, and Glycated Hemoglobin A1c

#### Cohort Characteristics

In the creatine kinase cohort, 496,821 participants were included for ALS and 497,026 for FTD (Supplementary Table S15). Among these, there were 438 ALS diagnoses (0.09%) and 142 FTD diagnoses (0.03%) during follow‐up. The median age at sampling differed: 54.4 years (interquartile range [IQR] = 51.1–71.2) for ALS and 65.7 years (IQR = 50.2–70.1) for FTD. Median follow‐up times were 8.8 (IQR = 6.2–12.7) years for ALS and 9.7 (IQR = 6.8–12.7) years for FTD.

The creatinine cohort consisted of over 4.6 million participants for both outcomes, with 2,695 ALS cases (0.06%) and 781 FTD cases (0.02%) identified (Supplementary Table S16). Participants diagnosed with ALS had a median sampling age of 62.3 years (IQR = 54.7–69.2 years), whereas those with FTD had a median sampling age of 62.9 years (IQR = 54.7–69.7 years). Follow‐up durations were 9.4 years (IQR = 6.4–13.4 years) for ALS and 10.5 years (IQR = 6.7–14.6 years) for FTD.

Last, the HbA1c cohort included 2,902,453 participants for ALS and 2,902,951 for FTD (Supplementary Table S17). There were 989 ALS cases (0.03%) and 368 FTD cases (0.01%) diagnosed. The median sampling age was 65.4 years (IQR = 57.1–72.1 years) for ALS and 67 years (IQR = 58.5–73 years) for FTD. The follow‐up times were shorter in this cohort, with medians of 6.5 years (IQR = 5.1–9.3 years) for ALS and 7 years (IQR = 5.2–9.7 years) for FTD.

#### Association with Amyotrophic Lateral Sclerosis and Frontotemporal Dementia

**FIGURE 4:**
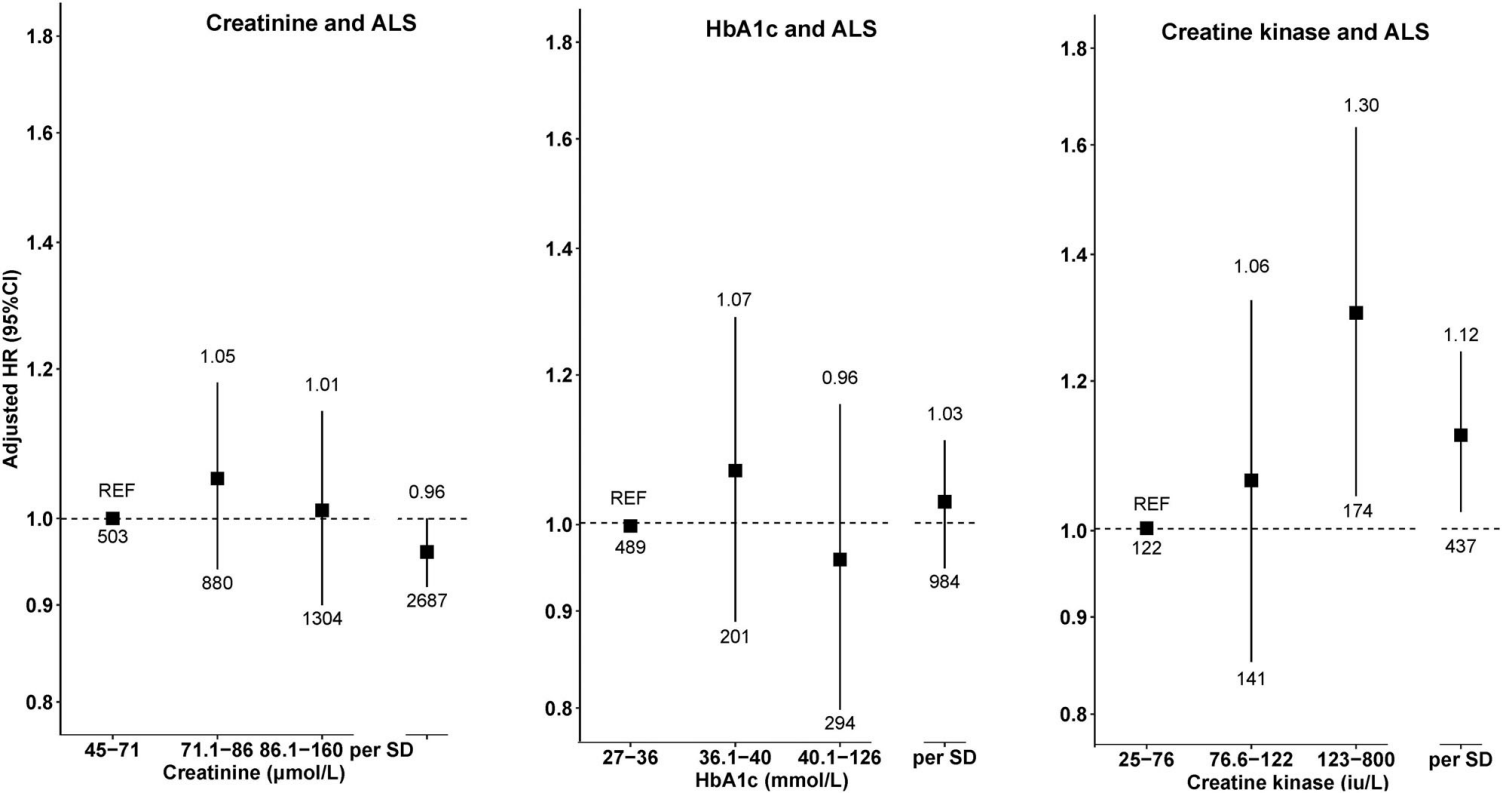
Association of the first measurement of creatinine, creatine kinase, HbA1c with the incidence of ALS. The numbers at the end of the lower and upper CI indicate, the total number of events and the effect estimate, respectively. ALS = amyotrophic lateral sclerosis; CI = confidence interval; HbA1c = hemoglobin A1c.

Creatine kinase demonstrated a linear association with increased ALS risk (HR_per 1‐SD increase_ = 1.12, 95% CI = 1.02–1.22, n_events_ = 437; Fig 4 and Supplementary Table S18). Compared with people in the lowest tertile of creatine kinase levels (25–76 log_2_ iu/L), those in the highest tertile (123–800 log_2_ iu/L) had a significantly higher risk of ALS (HR = 1.30, 95% CI = 1.04–1.63), suggesting a dose–response association (P_trend_ = 0.019). No associations were observed between creatinine or HbA1c and ALS risk. Furthermore, no evidence of sex or age effect modification was found in the association among creatine kinase, creatinine, or HbA1c levels and ALS risk (Supplementary Table S19).

**FIGURE 5:**
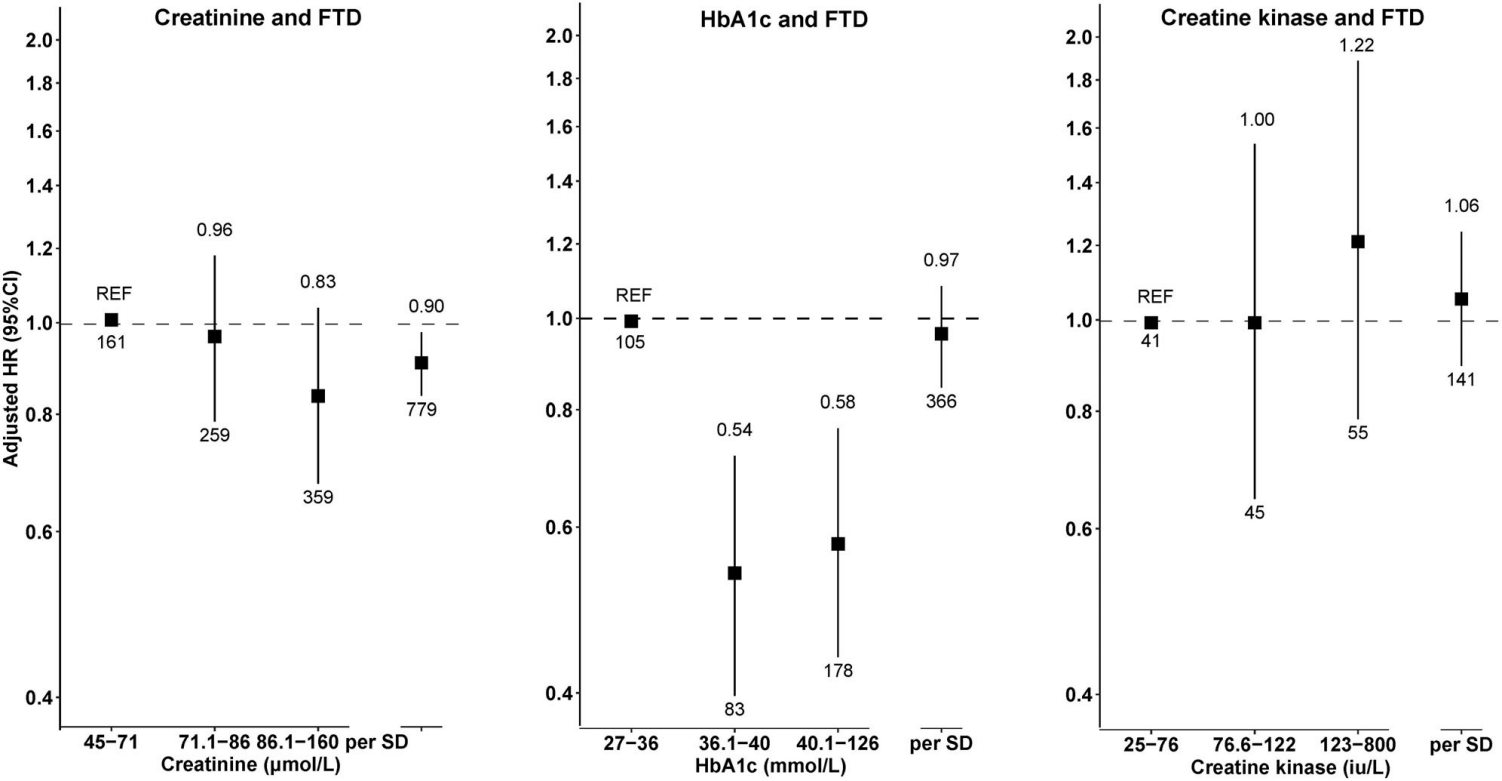
Association of the first measurement of creatinine, creatine kinase, HbA1c with the incidence of FTD. The numbers at the end of the lower and upper CI indicate, the total number of events and the effect estimate, respectively. CI = confidence interval; FTD = frontotemporal dementia; HbA1c = hemoglobin A1c.

Higher creatinine levels were linearly associated with a decreased risk of FTD (HR_per 1‐SD_ = 0.90, 95% CI = 0.83–0.97, n_events_ = 779, P_trend_ = 0.072; Fig 5 and Supplementary Table S20).

Although HbA1c did not show an association with FTD when modeled as a continuous variable, restricted cubic spline modeling revealed a nonlinear association between HbA1c and FTD (P_non‐linearity_ = 0.006; Supplementary Fig S2), suggesting that both very low and very high levels of HbA1c may be associated with an increased risk, whereas moderate levels are associated with lower risk.

The subgroup analyses showed that the associations of creatine kinase, creatinine, or HbA1c with FTD risk were consistent across age and sex groups, with no evidence of effect modification (Supplementary Table S21). The sensitivity analyses did not alter the above findings (Supplementary Tables S22–S25).

### Mendelian Randomization

#### Creatinine, HbA1c, Amyotrophic Lateral Sclerosis, and Frontotemporal Dementia

**FIGURE 6:**
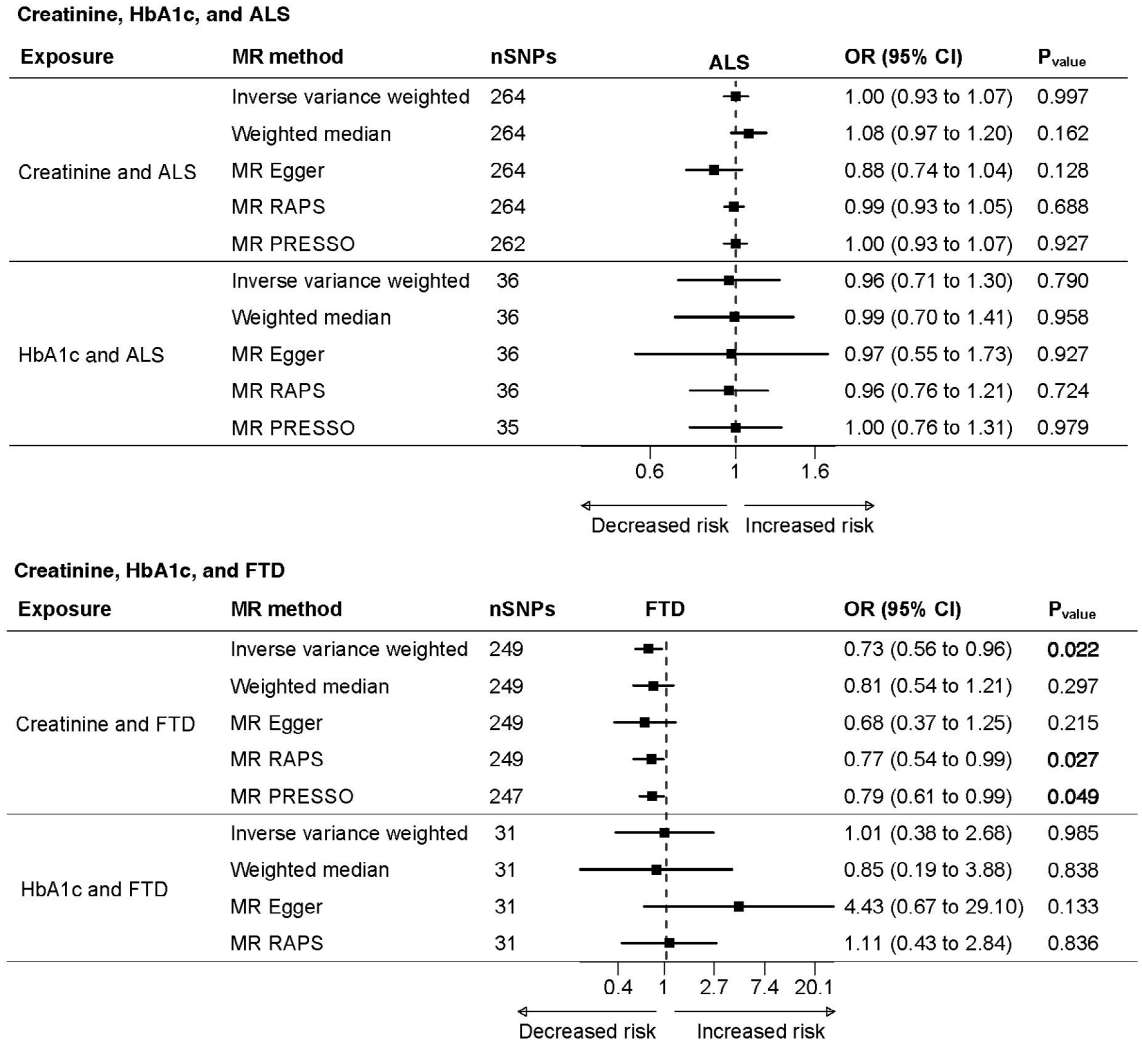
Association among genetically predisposed levels of creatinine, HbA1c, ALS and FTD according to MR. ALS = amyotrophic lateral sclerosis; FTD = frontotemporal dementia; HbA1c = hemoglobin A1c; MR = Mendelian randomization.

Consistent with findings in the above cohort, the MR analysis did not find evidence of an association between genetically predicted levels of creatinine or HbA1c and ALS (Fig 6 and Supplementary Table S26). However, higher genetically predicted creatinine levels were associated with a decreased FTD risk (odds ratio [OR] inverse variance weighted _(IVW), per 1‐SD_ = 0.73, 95% CI = 0.56–0.96; see Supplementary Table S26). MR robust adjusted profile score (RAPS) findings were similar (OR_MR‐RAPS, per 1‐SD increase_ = 0.77, 95% CI = 0.54–1.00), and all other sensitivity analyses were directionally consistent with the IVW results, although some estimates did not reach statistical significance, likely due to lower statistical power. MR‐PRESSO identified 2 outlier single nucleotide polymorphisms (SNPs), and a reanalysis excluding these SNPs remained consistent with the primary findings. No linear association was observed between genetically predicted HbA1c levels and FTD. Finally, the MR‐Egger intercept indicated no evidence of horizontal pleiotropy.

## Discussion

This study examined the associations of 7 blood biomarkers with the risk of developing ALS and FTD in a large real‐world cohort, including over 4 million individuals (up to 2,695 ALS and 781 FTD events) spanning over 25 years of data collection. To triangulate the observational findings, a 2‐sample MR analysis was conducted to explore potential genetic associations. The study identified 2 key findings. Higher LDL‐C and total cholesterol levels were associated with an increased risk of ALS, with the association for total cholesterol being particularly pronounced in individuals aged 60 years and older. Higher creatinine levels were associated with a reduced risk of FTD, with MR analysis providing supportive genetic evidence for this association. Additional findings were of an association between elevated creatine kinase levels and ALS risk and a nonlinear association between HbA1c levels and FTD risk. Notably, despite their genetic, pathological and clinical overlaps, distinct prediagnostic metabolic alterations were identified in those developing ALS and FTD.

The finding of an association between elevated LDL‐C and total cholesterol levels with an increased risk of ALS in this extremely large cohort recapitulates previous research, including MR‐based analysis, further highlighting the potential role of blood lipid biomarkers in ALS risk. A population‐based cohort study^11^ reported an association between higher LDL‐C levels and ALS risk but no significant association for total cholesterol and ALS risk, despite finding a magnitude of effect identical to this study (HR_per 1‐SD_ = 1.06). The discrepancy relating to total cholesterol is likely explained by the lower statistical power of that study, which included 567 ALS events, compared with 2,463 events in this study. Consistent with our estimates, a Norwegian cohort study of people aged 35 to 50 years found that each mmol/L increase in LDL‐C and total cholesterol was associated with a 7% and 18% increase in ALS risk, respectively.^20^ Genetic epidemiological studies support a genetic association between blood cholesterol levels and ALS risk.^15^, ^21^, ^22^ MR analyses, using progressively stronger and larger genetic instruments from successive releases of the same consortium genomewide association study (GWAS), have consistently supported a role for LDL‐C, whereas evidence for total cholesterol has been less consistent. These associations have remained consistent across sensitivity analyses assessing violations of MR assumptions, including horizontal pleiotropy, between‐instrument heterogeneity, and influence from outlier variants. Complementary analysis, using a polygenic score approach, demonstrates higher genetically predicted LDL‐C and total cholesterol levels in people with ALS, providing additional genetic evidence for an association between lipid levels and ALS risk.^21^ Key limitations include the restricted study population, limiting generalizability, and the reliance on exposure and outcome GWAS with overlapping samples, reducing the independence of findings across studies.

Existing literature has been somewhat conflicting in relation to HDL‐C, with studies identifying no association, an association between higher HDL‐C and lower ALS risk or, conversely, lower HDL‐C and lower ALS risk.^11^, ^12^, ^20^, ^23^ The findings of this study are consistent with genetic epidemiological analyses, which have failed to show evidence of a potential causal role for HDL‐C and triglycerides in ALS risk.^15^, ^22^, ^24^

No prior observational studies have investigated the relationship between lipid biomarkers and the risk of FTD, leaving our findings without direct comparison in the literature. However, a previous MR study^15^ examined genetically predicted levels of 4 lipid biomarkers and found evidence of an association of higher apolipoprotein B levels (but not LDL‐C, despite its high degree of correlation with apolipoprotein B) with FTD risk.^15^ This discrepancy might be a consequence of the lower power of both observational studies of FTD when compared to ALS (effect estimates in this study were similar to those of ALS albeit not statistically significant) or the inability of in‐life observational studies to separate pathological subtypes of FTD which could have differing etiological contributors.^4^, ^25^ Alternatively, this could indicate that lipoprotein particle number (captured by apolipoprotein B) may be more relevant to FTD pathophysiology than cholesterol content (captured by LDL‐C) and highlights the importance of considering different lipid biomarkers, potentially providing complementary information about lipid metabolism and disease risk. Due to the nature of the current study utilizing routinely collected primary care data, analysis of apolipoprotein levels was not possible.

Lipids are essential components of the central nervous system, playing critical roles in its normal functioning, which has spurred considerable interest in understanding their role in neurodegenerative diseases.^26^ Beyond ALS, disrupted cholesterol metabolism has also been implicated in conditions such as Alzheimer's, Parkinson's, and Huntington's diseases, highlighting lipid pathways as potential contributors to neurodegeneration more generally.^27^ Most cholesterol in the brain is synthesized locally, with minimal transfer from circulating cholesterol,^28^ raising questions about how blood lipid levels relate to neurodegenerative disease risk. Potential mechanisms include the impact of oxidized cholesterol species,^29^ which may mediate toxicity in neurons, or the transport of cholesterol derivatives between the brain and blood. Additionally, cholesteryl esters,^30^ which help manage excess cholesterol, might contribute to oxidative stress within neurons. Lipid levels could also be markers of more intricate synaptic changes during neurodegeneration,^31^ reflecting underlying neuronal signaling and connectivity alterations. Alternatively, changes in the cerebral vasculature might mediate the effects of cholesterol levels on neurodegenerative disease risk or indicate underlying presymptomatic metabolic derangements.^32^, ^33^ The lack of an association between cholesterol levels and FTD risk indicates that the effect of lipids is not a nonspecific alteration in neurodegenerative disease risk.

Several observational studies have reported lower serum creatinine levels in people with ALS,^34^, ^35^, ^36^, ^37^, ^38^ thought to indicate a loss of muscle mass occurring due to denervation in ALS. This is supported by the lack of association between creatinine levels years before diagnosis and the risk of ALS in the present study.

Beyond diagnostic associations, creatinine has also emerged as a strong prognostic biomarker in ALS. Higher baseline creatinine levels at diagnosis have been consistently associated with longer survival and better functional status.^39^, ^40^ Moreover, longitudinal studies demonstrate that faster declines in creatinine over follow‐up mirror accelerated Amyotrophic Lateral Sclerosis Functional Rating Scale‐Revised (ALSFRS‐R) deterioration and are associated with markedly shorter survival.^41^ Interestingly, however, lower creatinine levels were associated with an increased risk of FTD. Creatinine, a byproduct of creatine metabolism, supports neuronal energy homeostasis, with higher levels potentially counteracting energy deficits observed in FTD.^42^ Greater muscle mass, associated with elevated creatinine, is linked to better systemic health, reduced inflammation, and improved insulin sensitivity, which may mitigate neurodegeneration.^42^ Creatine also exhibits neuroprotective properties, including reducing oxidative stress and neuroinflammation, key processes in FTD.^42^ Reverse causation is possible, as early muscle wasting (sarcopenia) in prodromal FTD may lower creatinine levels, mimicking a protective effect, although this might be expected to be more likely in ALS due to prominent muscle wasting from denervation and is mitigated by the exclusion of people diagnosed with FTD within 4 years of biomarker measurement.^43^ However, the association between creatinine and FTD risk was also confirmed through MR, which is less susceptible to confounding and reverse causality as it relies on genetic variants randomly assorted at conception. This supports a potential causal role of creatinine in FTD risk, warranting further investigation.^44^, ^45^

Specific research directly linking HbA1c levels to FTD is limited. Most studies have focused on the association between HbA1c levels and overall dementia risk, particularly in individuals with diabetes.^46^, ^47^ Similar to our findings, a cohort study found a protective association between moderate HbA1c exposure (6%–8%) and dementia risk.^46^ The protective association of moderate HbA1c levels may reflect optimal metabolic health and resilience to neurodegeneration, whereas higher HbA1c levels could signal systemic dysfunction, contributing to neuroinflammation and vascular damage that accelerate FTD pathology.^48^, ^49^, ^50^ Given the distinct pathophysiology of FTD compared with other forms of dementia, more targeted research is needed to determine if there is a specific association between HbA1c levels and the development or progression of FTD.

Higher creatine kinase levels showed a linear association with increased ALS risk. Although the interaction test between sex and the association of creatine kinase and ALS risk did not reach statistical significance, the stratified estimates suggest a potentially stronger association between creatinine kinase and ALS risk in men than women. It is worth noting that, in symptomatic ALS, higher creatine kinase levels are observed in people with limb onset of symptoms, who are more frequently male patients, when compared to bulbar onset of symptoms, which is more common in women. Although this might explain these differences, data concerning site of symptom onset is not available within this dataset. The mechanism underlying creatine kinase elevation in ALS remains poorly understood. Current hypotheses include that disturbances in muscle energy metabolism may enhance endogenous ATP activity within mitochondria, leading to upregulated creatine kinase expression to supply an energy substrate,^51^ or that elevated creatine kinase levels may reflect greater muscle cell membrane permeability, attributed to denervated muscles and historical myopathic changes observed in people with ALS.^52^ Although excluding people developing ALS within 4 years of biomarker sampling should reduce the risk of reverse causality, creatine kinase levels are not measured as part of routine health screening but in relation to symptomatology, meaning that this risk is not fully mitigated. Due to the lack of a suitable GWAS of creatine kinase levels, it was not possible to further examine the potential genetic association of creatine kinase levels with ALS risk.

Several limitations should be considered when interpreting our findings. Potential inaccuracies in diagnostic coding are likely to have occurred, although HES data and linked GP records remain a robust method for maximizing case ascertainment.^53^ A systematic review of routinely collected health care data for motor neuron disease (MND) reported positive predictive values ranging from 55 to 92% and sensitivities of 75 to 93%, with UK‐based studies demonstrating the highest accuracy.^54^ When 81 general practitioner (GP)‐recorded ALS diagnoses were reviewed by a neurologist in the Clinical Practice Research Datalink, the diagnosis was confirmed in 85% of cases.^55^ Subsequent studies have confirmed the reliability of MND coding,^56^ and, in the current study, 81% of patients with ALS were identified by at least 2 data sources, which supports the reliability of this method of identifying cases. Reverse causality is possible. In ALS, biochemical evidence of active neurodegeneration begins 6 to 12 months before the first symptoms and diagnosis is obtained within 1 year of symptom onset in most cases.^57^, ^58^, ^59^ In FTD, the pre‐diagnostic period is likely to be longer; excluding people with less than 4 years of follow‐up following biomarker measurement should be adequate to negate this risk, with MR analysis providing independent evidence of causality. Although we adjusted for several potential confounders, the possibility of unmeasured or residual confounding cannot be entirely excluded. Younger‐onset events are under‐represented, and individuals with earlier and more frequent blood tests—often those with poorer health—may have higher lipid or HbA1c levels and longer follow‐up, increasing their likelihood of an ALS or FTD diagnosis. This surveillance bias could inflate associations, although it reflects the best available data. We were unable to account for temporal changes in covariates such as smoking and BMI over follow‐up, which may have introduced residual confounding. Finally, a limitation in analyzing primary care data is the lack of genetic data for people with ALS and FTD, where the impact of risk‐modifying measures has the greatest potential for impact.

## Conclusions

This study provides evidence of differing prediagnostic metabolic profiles in those going on to develop ALS and FTD. Beyond confirming the observational associations of LDL and total cholesterol with ALS risk and indicating a potential relevance of creatinine and HbA1c levels for FTD risk, the findings suggest that systemic metabolic differences may be associated with the development of these related neurodegenerative diseases. Further work in those at risk of monogenetic forms of ALS and FTD is necessary to determine whether these factors might be predictive of the phenotype of disease during its clinically manifest stage, and whether they influence penetrance as a step toward prevention. In addition, future studies can evaluate potential mediation pathways in the lipid–ALS association.

## Author Contributions

C.V.C., C.A.C.C., J.H.C., M.R.T., and A.G.T. contributed to conception and design of the study; C.V.C. and J.H.C. contributed to the acquisition and analysis of data; C.V.C. contributed to drafting the text or preparing the figures.

## Potential Conflicts of Interest

Nothing to report.

## Supporting information


**Supplementary Data S1** Supporting Information

## Data Availability

Only C.V.C. had full access to the data during the study, in accordance with the relevant license agreements, to guarantee the confidentiality of patients’ personal and health information. Information on access to the QResearch data is available on the QResearch website (http://www.qresearch.org/).
