# Supplementary material for: Amyotrophic Lateral Sclerosis and Frontotemporal Dementia Have Distinct Prediagnostic Blood Biochemical Profiles
Source: Ann Neurol. 2025 Oct 30;99(4):844–56. doi: 10.1002/ana.78082 (PMC13011780; doi:10.1002/ana.78082)
Supplement: Supplementary file 1 — Supplementary Data S1 Supporting Information [file ANA-99-844-s001.docx]

**Amyotrophic lateral sclerosis and frontotemporal dementia have distinct prediagnostic blood biochemical profiles**

Christos V. Chalitsios, Jiali Gao, Carol A.C. Coupland, Julia Hippisley Cox, Martin R. Turner, Alexander G. Thompson

**Supplements**

[**eTable 1**. Read/SNOMED and ICD-10 codes used to identify amyotrophic lateral sclerosis and frontotemporal dementia. 5](#_Toc191651629)

[**eTable 2**. Baseline characteristics of people with at least one HDL-c measurement with and without ALS/ with and without FTD at least four years after the first HDL-c measurement. 6](#_Toc191651630)

[**eTable 3**. Baseline characteristics of people with at least one LDL-c measurement with and without ALS/with and without FTD at least four years after the first LDL-c measurement. 7](#_Toc191651631)

[**eTable 4**. Baseline characteristics of people with at least one total cholesterol measurement with and without ALS/ with and without FTD at least four years after the first total cholesterol measurement. 8](#_Toc191651632)

[**eTable 5**. Baseline characteristics of people with at least one triglycerides measurement with and without ALS/ with and without FTD at least four years after the first triglycerides measurement. 9](#_Toc191651633)

[**eTable 6**. Association of the first measurement of lipid blood biomarkers with the incidence of amyotrophic lateral sclerosis. 10](#_Toc191651634)

[**eTable 7**. Association (per 1-SD increase) of the first measurement of lipid blood biomarkers with the incidence of amyotrophic lateral sclerosis stratified by sex, median age, and lipid-lowering treatment. 11](#_Toc191651635)

[**eTable 8**. Association of the first measurement of lipid blood biomarkers with the incidence of frontotemporal dementia. For each biomarker, the first row represents its association modelled continuously (per 1-SD increase), while subsequent rows evaluate the association categorically across tertiles, with the first tertile serving as the reference group. 12](#_Toc191651636)

[**eTable 9**. Association (per 1-SD increase) of the first measurement of lipid blood biomarkers with the incidence of frontotemporal dementia stratified by sex, median age, and lipid-lowering treatment. 13](#_Toc191651637)

[**eTable 10.** Association of the first measurement of lipid blood biomarkers with the incidence of amyotrophic lateral sclerosis after excluding those with a history of FTD diagnosis. For each biomarker, the first row represents its association modelled continuously (per 1-SD increase), while subsequent rows evaluate the association categorically across tertiles, with the first tertile serving as the reference group. 15](#_Toc191651638)

[**eTable 11**. Association of the first measurement of lipid blood biomarkers with the incidence of frontotemporal dementia after excluding those with a history of ALS diagnosis. For each biomarker, the first row represents its association modelled continuously (per 1-SD increase), while subsequent rows evaluate the association categorically across tertiles, with the first tertile serving as the reference group. 15](#_Toc191651639)

[**eTable 12**. Association of the first measurement of LDL-c (considering additionally greater levels than 10 mmol/L) with the incidence of amyotrophic lateral (ALS) sclerosis and frontotemporal dementia (FTD). The first row represents its continuous scale measurement (per 1-SD increase), and subsequent rows categorise LDL-c into tertiles, with the first tertile used as the reference category. 16](#_Toc191651640)

[**eTable 13**. Association of the first measurement of lipid blood biomarkers with the incidence of amyotrophic lateral sclerosis adjusting for further covariates. For each biomarker, the first row represents its association modelled continuously (per 1-SD increase), while subsequent rows evaluate the association categorically across tertiles, with the first tertile serving as the reference group. 16](#_Toc191651641)

[**eTable 14**. Association of the first measurement of lipid blood biomarkers with the incidence of frontotemporal dementia adjusting for further covariates. For each biomarker, the first row represents its association modelled continuously (per 1-SD increase), while subsequent rows evaluate the association categorically across tertiles, with the first tertile serving as the reference group. 17](#_Toc191651642)

[**eTable 15**. Baseline characteristics of people with at least one creatine kinase measurement with and without ALS/ with and without FTD at least four years after the first creatine kinase measurement. 18](#_Toc191651643)

[**eTable 16**. Baseline characteristics of people with at least one creatinine measurement with and without ALS/ with and without FTD at least four years after the first creatinine measurement. 19](#_Toc191651644)

[**eTable 17**. Baseline characteristics of people with at least one HbA1c measurement with and without ALS/ with and without FTD at least four years after the first HbA1c measurement. 20](#_Toc191651645)

[**eTable 18**. Association of the first measurement of creatinine, creatine kinase, and HbA1c with the incidence of amyotrophic lateral sclerosis. For each biomarker, the first row represents its association modelled continuously (per 1-SD increase), while subsequent rows evaluate the association categorically across tertiles, with the first tertile serving as the reference group. 21](#_Toc191651646)

[**eTable 19**. Association (per 1-SD increase) of the first measurement of creatinine, creatine kinase, and HbA1c with the incidence of amyotrophic lateral sclerosis stratified by sex and median age. 21](#_Toc191651647)

[**eTable 20**. Association of the first measurement of creatinine, creatine kinase, and HbA1c with the incidence of frontotemporal dementia. For each biomarker, the first row represents its association modelled continuously (per 1-SD increase), while subsequent rows evaluate the association categorically across tertiles, with the first tertile serving as the reference group. 22](#_Toc191651648)

[**eTable 21**. Association (per 1-SD increase) of the first measurement of creatinine, creatine kinase, and HbA1c with the incidence of frontotemporal dementia stratified by sex and median age. 23](#_Toc191651649)

[**eTable 22.** Association of the first measurement of creatinine, creatine kinase, and HbA1c with the incidence of amyotrophic lateral sclerosis after excluding those with a history of FTD diagnosis. For each biomarker, the first row represents its association modelled continuously (per 1-SD increase), while subsequent rows evaluate the association categorically across tertiles, with the first tertile serving as the reference group. 24](#_Toc191651650)

[**eTable 23**. Association of the first measurement of creatinine, creatine kinase, and HbA1c with the incidence of frontotemporal dementia after excluding those with a history of ALS diagnosis. For each biomarker, the first row represents its association modelled continuously (per 1-SD increase), while subsequent rows evaluate the association categorically across tertiles, with the first tertile serving as the reference group. 24](#_Toc191651651)

[**eTable 24**. Association of the first measurement of creatinine, creatine kinase, and HbA1c with the incidence of amyotrophic lateral sclerosis adjusting for further covariates. For each biomarker, the first row represents its association modelled continuously (per 1-SD increase), while subsequent rows evaluate the association categorically across tertiles, with the first tertile serving as the reference group. 25](#_Toc191651652)

[**eTable 25**. Association of the first measurement of creatinine, creatine kinase, and HbA1c with the incidence of frontotemporal dementia adjusting for further covariates. For each biomarker, the first row represents its association modelled continuously (per 1-SD increase), while subsequent rows evaluate the association categorically across tertiles, with the first tertile serving as the reference group. 25](#_Toc191651653)

[**eTable 26**. Association (per 1-SD increase) of genetically predicted levels of creatinine and HbA1c with amyotrophic lateral sclerosis (ALS) and frontotemporal dementia (FTD) based on the two-sample Mendelian randomisation analysis. 26](#_Toc191651654)

[**eFigure 1**. Non-linear relationship between the lipid blood biomarkers and amyotrophic lateral sclerosis (ALS) using the Cox proportional models 12](#_Toc191651552)

[**eFigure 2**. Non-linear relationship between creatinine, creatine kinase, HbA1c and amyotrophic lateral sclerosis (ALS) using the Cox proportional models 20](#_Toc191651553)

## Supplementary methods - Mendelian randomisation

### Genetic variant selection

Independent genetic variants (linkage disequilibrium [LD] clumping threshold of r2 < 0.001, using a reference panel consisting of individuals of European ancestry from the 1,000 Genomes Project Consortium (1), within a 10,000 kb window) associated with creatinine and HbA1c at genome-wide significance (p < 5 × 10−8) were identified from a GWAS using data from the UK Biobank (n=363,228) (2) and from a GWAS meta-analysis of 50 cohorts in 123,665 Europeans (3). The F-statistic for each variant-trait association was calculated to evaluate instrument strength and potential violation of the first MR assumption (i.e. the IV must be associated with the exposure), and only the genetic variants with an F-statistic > 10 were included (4,5). Genetic instruments with an effect allele frequency ≥ 0.01 were included.

### Outcomes

The outcomes were ALS and FTD. For ALS, summary statistics were obtained from the largest available GWAS (6), including 27,205 cases and 110,881 controls of European ancestry from Project MinE. All patients with ALS were diagnosed and ascertained through specialised motor neuron diseases (MND) clinics, where they were diagnosed with ALS according to the (revised) El Escorial Criteria (7) by neurologists specialised in ALS. For FTD, summary statistics were obtained from the largest GWAS (8), including 2,154 cases and 4,308 controls of European ancestry. Patients diagnosed according to Neary criteria (9) with behavioural variant FTD, semantic dementia, progressive non-fluent aphasia, and FTD overlapping with motor neuron disease were included in the GWAS.

### Statistical analysis

The primary analysis was random-effects inverse variance–weighted (IVW) MR (10). To account for potential horizontal pleiotropy, several MR sensitivity analyses (MR-Egger (11), weighted median (12), and weighted mode (13)) were performed, each providing a valid MR estimate under different combinations of assumptions. To detect potential outlying IVs, we implemented the MR pleiotropy residual sum and outlier test (MR-PRESSO), which identifies and excludes outliers, applying a random-effects IVW model (14). In addition, MR, using a robust adjusted profile score (MR-RAPS) (15), was used to control for pleiotropy through a random effects model, considering the variance in instrument effect sizes. MR analysis was performed with R v4.3.1 using the “TwoSampleMR” and “mr.raps” packages.

**eTable 1**. Read/SNOMED and ICD-10 codes used to identify amyotrophic lateral sclerosis and frontotemporal dementia.

| **Condition** | **Coding system** | **Code** | **Description** |
| --- | --- | --- | --- |
| ALS | Read/SNOMED | F152 | Motor neuron disease |
| ALS | Read/SNOMED | F1520 | Amyotrophic lateral sclerosis |
| ALS | Read/SNOMED | F1521 | Progressive muscular atrophy |
| ALS | Read/SNOMED | F1522 | Progressive bulbar palsy |
| ALS | Read/SNOMED | F1523 | Pseudobulbar palsy |
| ALS | Read/SNOMED | F1524 | Primary lateral sclerosis |
| ALS | ICD-10 | G12.2 | Motor neuron disease |
| FTD | Read/SNOMED | F118 | Frontotemporal degeneration |
| FTD | Read/SNOMED | F111 | Pick’s disease |
| FTD | ICD-10 | F02.0 | Dementia in Pick disease |
| FTD | ICD-10 | G31.0 | Frontotemporal dementia |

ALS, Amyotrophic Lateral Sclerosis; FTD, Frontotemporal dementia; MND, Motor Neuron Disease

**eTable 2**. Baseline characteristics of people with at least one HDL-c measurement with and without ALS/ with and without FTD at least four years after the first HDL-c measurement.

|  | **People without ALS (n=3,752,384)** | **People with ALS (n=2,249)** | **People without FTD (n=3,754,363)** | **People with FTD (n=678)** |
| --- | --- | --- | --- | --- |
| **Follow-up time, median (IQR), yrs.** | 6.1 (6.2-13.8) | 8.8 (6.2-12.5) | 6.1 (6.2-13.8) | 9.6 (6.5-13.4) |
| **HDL-c, mmol/L** |  |  |  |  |
| Median (IQR) | 1.4 (1.1-1.7) | 1.4 (1.1-1.7) | 1.4 (1.1-1.7) | 1.4 (1.2-1.7) |
| Mean (SD) | 1.4 (0.4) | 1.5 (0.4) | 1.4 (0.4) | 1.4 (0.4) |
| **Age at sampling, median (IQR), yrs.** | 55.4 (45.5-66.4) | 63.2 (56.3-69.7) | 55.4 (45.5-66.4) | 63.2 (55.4-70.5) |
| **Sex, males** | 1,813,815 (48.3) | 1,282 (57) | 1,815,180 (48.3) | 381 (56.2) |
| **Townsend deprivation index** |  |  |  |  |
| Least deprived | 1,125,115 (30) | 861 (38.3) | 1,126,015 (30) | 216 (31.9) |
| - | 883,038 (23.5) | 583 (25.9) | 883,614 (23.5) | 167 (24.6) |
| - | 701,310 (18.7) | 333 (14.8) | 701,627 (18.7) | 122 (18) |
| - | 565,392 (15.1) | 270 (12) | 565,654 (15.1) | 92 (13.6) |
| Most deprived | 462,429 (12.3) | 195 (8.7) | 462,612 (12.3) | 78 (11.5) |
| Missing | 15,100 (0.4) | 7 (0.3) | 15,114 (0.4) | <5 |
| **Body mass index, mean (SD),** $\frac{\boldsymbol{kg}}{\boldsymbol{m}^{\boldsymbol{2}}}$ | 27.2 (5.5) | 27.8 (4.9) | 27.2 (5.5) | 27.4 (4.7) |
| Missing | 1,750,681 (46.6) | 1,033 (45.9) | 1,751,767 (46.7) | 304 (44.8) |
| **Smoking status** |  |  |  |  |
| Non-smokers | 1,270,071 (33.9) | 740 (32.9) | 1,270,824 (33.9) | 210 (31) |
| Ex-smokers | 732,657 (19.5) | 490 (21.8) | 733,141 (19.5) | 142 (20.9) |
| Current smokers | 583,689 (15.6) | 294 (13.1) | 583,945 (15.6) | 110 (16.2) |
| Missing | 1,165,967 (31.1) | 725 (32.2) | 1,166,726 (31.1) | 216 (21.9) |
| **Comorbidity (any before the index date)** |  |  |  |  |
| Diabetes | 247,957 (6.6) | 163 (7.3) | 248,094 (6.6) | 52 (7.7) |
| Cardiovascular diseases | 168,330 (4.5) | 183 (8.1) | 168,536 (4.5) | 41 (6.1) |
| Peripheral vascular disease | 17,284 (0.5) | 13 (0.6) | 17,300 (0.5) | <5 |
| Chronic kidney disease | 47,528 (1.3) | 32 (1.4) | 47,564 (1.3) | 11 (1.6) |
| Atrial fibrillation | 49,654 (1.3) | 32 (1.4) | 49,685 (1.3) | 11 (1.6) |
| Family history of MND | 93 (0.001) | <5 | 93 (0.001) | <5 |
| Family history of angina | 7,351 (0.2) | <5 | 7,353 (0.2) | <5 |
| **Lipid-lowering therapy use, ever** | 1,306,971 (34.8) | 845 (37.6) | 1,307,790 (34.8) | 264 (38.9) |

ALS, Amyotrophic Lateral Sclerosis; FTD, Frontotemporal dementia; MND, Motor Neuron Disease

All figures are presented as absolute numbers (percentage) unless otherwise specified.

Counts less than 5 are suppressed for confidentiality.

**eTable 3**. Baseline characteristics of people with at least one LDL-c measurement with and without ALS/with and without FTD at least four years after the first LDL-c measurement.

|  | **People without ALS (n=3,030,025)** | **People with ALS (n=1,898)** | **People without FTD (n=3,031,909)** | **People with FTD (n=573)** |
| --- | --- | --- | --- | --- |
| **Follow-up time, median (IQR), yrs.** | 9.5 (6.3-14.1) | 8.9 (6.2-12.4) | 9.5 (6.3-14.1) | 9.5 (6.5-13) |
| **LDL-c, mmol/L** |  |  |  |  |
| Median (IQR) | 3.1 (2.5-3.8) | 3.3 (2.6-3.9) | 3.1 (2.5-3.8) | 3.2 (2.5-3.9) |
| Mean (SD) | 3.2 (1) | 3.3 (1) | 3.2 (1) | 3.3 (1.1) |
| **Age at sampling, median (IQR), yrs.** | 56.1 (45.9-66.9) | 63.8 (56.5-70) | 56.1 (45.9-66.9) | 63.8 (56.4-70.6) |
| **Sex, males** | 1,464,323 (48.3) | 1,088 (57.3) | 1,465,455 (48.3) | 320 (55.9) |
| **Townsend deprivation index** |  |  |  |  |
| Least deprived | 901,588 (29.8) | 743 (39.2) | 902,353 (29.8) | 181 (31.6) |
| - | 703,229 (23.2) | 486 (25.6) | 703,694 (23.2) | 140 (24.4) |
| - | 570,814 (18.8) | 281 (14.8) | 571,090 (18.8) | 104 (18.2) |
| - | 463,940 (15.3) | 227 (12) | 464,155 (15.3) | 80 (14) |
| Most deprived | 379,124 (12.5) | 154 (8.1) | 379,278 (12.5) | 65 (11.3) |
| Missing | 11,330 (0.4) | 7 (0.4) | 11,339 (0.4) | <5 |
| **Body mass index, mean (SD),** $\frac{\boldsymbol{kg}}{\boldsymbol{m}^{\boldsymbol{2}}}$ | 28.1 (5.5) | 27.7 (4.9) | 28.1 (5.5) | 27.4 (4.8) |
| Missing | 1,353,543 (44.7) | 832 (43.8) | 1,354,396 (44.7) | 235 (41) |
| **Smoking status** |  |  |  |  |
| Non-smokers | 1,052,943 (34.8) | 639 (33.7) | 1,053,585 (34.8) | 186 (32.5) |
| Ex-smokers | 623,556 (20.6) | 433 (22.8) | 623,977 (20.6) | 133 (23.2) |
| Current smokers | 475,214 (15.7) | 258 (13.6) | 475,447 (15.7) | 101 (17.6) |
| Missing | 878,312 (29) | 568 (29.9) | 878,900 (29) | 153 (26.7) |
| **Comorbidity (any before the index date)** |  |  |  |  |
| Diabetes | 223,855 (7.4) | 139 (7.3) | 223,983 (7.4) | 49 (8.6) |
| Cardiovascular diseases | 160,624 (5.3) | 179 (9.4) | 160,819 (5.3) | 36 (6.3) |
| Peripheral vascular disease | 16,813 (0.6) | 10 (0.5) | 16,825 (0.6) | <5 |
| Chronic kidney disease | 50,366 (1.7) | 33 (1.7) | 50,398 (1.7) | 15 (2.6) |
| Atrial fibrillation | 45,945 (1.5) | 31 (1.6) | 45,979 (1.5) | 11 (1.9) |
| Family history of MND | 85 (0.001) | <5 | 85 (0.001) | <5 |
| Family history of angina | 5,082 (0.2) | <5 | 5,083 (0.2) | <5 |
| **Lipid-lowering therapy use, ever** | 1,112,500 (36.7) | 722 (38.04) | 1,113,193 (36.7) | 232 (40.5) |

ALS, Amyotrophic Lateral Sclerosis; FTD, Frontotemporal dementia; MND, Motor Neuron Disease

All figures are presented as absolute numbers (percentage) unless otherwise specified.

Counts less than 5 are suppressed for confidentiality.

**eTable 4**. Baseline characteristics of people with at least one total cholesterol measurement with and without ALS/ with and without FTD at least four years after the first total cholesterol measurement.

|  | **People without ALS (n=3,951,024)** | **People with ALS (n=2,471)** | **People without FTD (n=3,953,499)** | **People with FTD (n=726)** |
| --- | --- | --- | --- | --- |
| **Follow-up time, median (IQR), yrs.** | 9.3 (6.3-14.5) | 9.2 (6.4-13.1) | 9.3 (6.3-14.5) | 10.3 (6.8-14.6) |
| **Total cholesterol, mmol/L** |  |  |  |  |
| Median (IQR) | 5.3 (4.5-6) | 5.5 (4.8-6.2) | 5.3 (4.5-6) | 5.5 (4.7-6.3) |
| Mean (SD) | 5.3 (1.1) | 5.5 (1.1) | 5.3 (1.1) | 5.5 (1.1) |
| **Age at sampling, median (IQR), yrs.** | 55.1 (45-66.1) | 62.7 (52.2-69.3) | 55.1 (45-66.1) | 62.7 (54.9-69.7) |
| **Sex, males** | 1,908,087 (48.3) | 1,418 (57.4) | 1,909,586 (48.3) | 408 (56.2) |
| **Townsend deprivation index** |  |  |  |  |
| Least deprived | 1,181,320 (29.9) | 932 (37.7) | 1,182,299 (29.9) | 227 (31.3) |
| - | 928,085 (23.5) | 627 (25.4) | 928,705 (23.5) | 180 (24.8) |
| - | 739,495 (18.7) | 390 (15.8) | 739,876 (18.7) | 130 (17.9) |
| - | 595,160 (15.1) | 293 (11.9) | 595,435 (15.1) | 100 (13.8) |
| Most deprived | 491,232 (12.4) | 221 (8.9) | 491,440 (12.4) | 86 (11.9) |
| Missing | 15,732 (0.4) | 8 (0.2) | 15,744 (0.4) | <5 |
| **Body mass index, mean (SD),** $\frac{\boldsymbol{kg}}{\boldsymbol{m}^{\boldsymbol{2}}}$ | 27.9 (5.5) | 27.7 (4.9) | 27.9 (5.5) | 27.4 (4.7) |
| Missing | 2,034,318 (51.5) | 1,321 (53.4) | 2,035,668 (51.5) | 384 (59.9) |
| **Smoking status** |  |  |  |  |
| Non-smokers | 1,243,323 (31.5) | 726 (29.4) | 1,244,092 (31.5) | 192 (16.5) |
| Ex-smokers | 693,151 (17.5) | 449 (18.2) | 693,596 (17.5) | 132 (18.2) |
| Current smokers | 587,757 (14.9) | 307 (12.4) | 588,046 (14.9) | 106 (14.6) |
| Missing | 1,426,793 (36.1) | 989 (40) | 1,427,765 (36.1) | 296 (40.8) |
| **Comorbidity (any before the index date)** |  |  |  |  |
| Diabetes | 225,189 (5.7) | 151 (6.1) | 225,333 (5.7) | 49 (6.7) |
| Cardiovascular diseases | 148,593 (3.8) | 159 (6.4) | 148,775 (3.8) | 35 (4.8) |
| Peripheral vascular disease | 14,274 (0.4) | 11 (0.5) | 14,288 (0.4) | <5 |
| Chronic kidney disease | 35,033 (0.9) | 19 (0.8) | 35,058 (0.9) | 5 (0.7) |
| Atrial fibrillation | 44,555 (1.1) | 21 (0.9) | 44,579 (1.1) | 9 (1.2) |
| Family history of MND | 91 (0.001) | <5 | 91 (0.001) | <5 |
| Family history of angina | 6,741 (0.2) | <5 | 6,743 (0.2) | <5 |
| **Lipid-lowering therapy use, ever** | 1,336,633 (33.8) | 886 (35.9) | 1,337,469 (33.8) | 269 (37) |

ALS, Amyotrophic Lateral Sclerosis; FTD, Frontotemporal dementia; MND, Motor Neuron Disease.

All figures are presented as absolute numbers (percentage) unless otherwise specified.

Counts less than 5 are suppressed for confidentiality.

**eTable 5**. Baseline characteristics of people with at least one triglycerides measurement with and without ALS/ with and without FTD at least four years after the first triglycerides measurement.

|  | **People without ALS (n=3,271,929)** | **People with ALS (n=2,119)** | **People without FTD (n=3,274,043)** | **People with FTD (n=624)** |
| --- | --- | --- | --- | --- |
| **Follow-up time, median (IQR), yrs.** | 9.5 (6.3-14.5) | 9.1 (6.3-12.9) | 9.5 (6.3-14.5) | 9.9 (6.7-10.2) |
| **Triglycerides, mmol/L** |  |  |  |  |
| Median (IQR) | 1.3 (0.9-1.9) | 1.4 (1-2) | 1.3 (0.9-1.9) | 1.3 (1-2) |
| Mean (SD) | 1.6 (0.9) | 1.6 (0.9) | 1.6 (0.9) | 1.6 (0.9) |
| **Age at sampling, median (IQR), yrs.** | 55.8 (45.7-66.8) | 63.2 (56.1-69.7) | 55.8 (45.7-66.8) | 63.2 (55.5-70.2) |
| **Sex, males** | 1,588,182 (48.5) | 1,205 (56.9) | 1,589,453 (48.5) | 347 (55.6) |
| **Townsend deprivation index** |  |  |  |  |
| Least deprived | 956,358 (29.2) | 804 (37.9) | 957,188 (29.2) | 193 (30.9) |
| - | 756,739 (23.1) | 530 (25) | 757,247 (23.1) | 151 (24.2) |
| - | 617,258 (18.9) | 329 (15.5) | 617,593 (18.9) | 113 (18.1) |
| - | 510,368 (15.6) | 256 (12.1) | 510,608 (15.6) | 89 (14.3) |
| Most deprived | 419,088 (12.8) | 192 (9.1) | 419,279 (12.8) | 75 (12) |
| Missing | 12,118 (0.4) | 8 (0.4) | 12,128 (0.4) | <5 |
| **Body mass index, mean (SD),** $\frac{\boldsymbol{kg}}{\boldsymbol{m}^{\boldsymbol{2}}}$ | 28.1 (5.5) | 27.8 (4.9) | 28.1 (5.5) | 27.5 (4.8) |
| Missing | 1,564,369 (47.8) | 1,020 (48.1) | 1,565,403 (47.8) | 284 (45.5) |
| **Smoking status** |  |  |  |  |
| Non-smokers | 1,081,531 (33.1) | 660 (31.2) | 1,082,216 (33.1) | 186 (29.8) |
| Ex-smokers | 628,931 (19.2) | 430 (20.3) | 629,362 (19.2) | 127 (20.4) |
| Current smokers | 506,351 (15.5) | 279 (13.2) | 506,597 (15.5) | 103 (16.5) |
| Missing | 1,055,116 (32.2) | 750 (35.4) | 1,055,868 (32.2) | 208 (33.3) |
| **Comorbidity (any before the index date)** |  |  |  |  |
| Diabetes | 222,860 (6.8) | 141 (6.7) | 222,994 (6.8) | 51 (8.2) |
| Cardiovascular diseases | 158,205 (4.8) | 170 (8) | 158,400 (4.8) | 37 (5.9) |
| Peripheral vascular disease | 16,034 (0.5) | 12 (0.6) | 16,049 (0.5) | <5 |
| Chronic kidney disease | 44,350 (1.4) | 24 (1.1) | 44,350 (1.4) | 10 (1.6) |
| Atrial fibrillation | 45,496 (1.4) | 27 (1.3) | 45,496 (1.4) | 8 (1.3) |
| Family history of MND | 90 (0.001) | <5 | 90 (0.001) | <5 |
| Family history of angina | 5,278 (0.2) | <5 | 5,279 (0.2) | <5 |
| **Lipid-lowering therapy use, ever** | 1,191,726 (36.4) | 808 (38.1) | 1,192,513 (36.4) | 247 (39.6) |

ALS, Amyotrophic Lateral Sclerosis; FTD, Frontotemporal dementia; MND, Motor Neuron Disease

All figures are presented as absolute numbers (percentage) unless otherwise specified.

Counts less than 5 are suppressed for confidentiality.

**eTable 6**. Association of the first measurement of lipid blood biomarkers with the incidence of amyotrophic lateral sclerosis. For each biomarker, the first row represents its association modelled continuously (per 1-SD increase), while subsequent rows evaluate the association categorically across tertiles, with the first tertile serving as the reference group.

| **Biomarker** | **N** | **No of events** | **Adjusted^1^**  **HR (95%CI)** | **P-value** |
| --- | --- | --- | --- | --- |
| **LDL-c (mmol/L)** | 3,020,586 | 1,891 | 1.07 (1.02-1.11) | 0.003 |
| 1-2.60 | 932,796 | 491 | Reference |  |
| 2.61-3.50 | 1,055,833 | 646 | 1.11 (0.99-1.24) |  |
| 3.51-10 | 1,031,957 | 754 | 1.16 (1.04-1.30) |  |
| *p* for trend |  |  | 0.008 |  |
| **HDL-c (mmol/L)** | 3,739,526 | 2,242 | 0.99 (0.95-1.04) | 0.812 |
| 0.50-1.20 | 1,300,860 | 768 | Reference |  |
| 1.21-1.56 | 1,181,106 | 728 | 0.98 (0.88-1.08) |  |
| 1.57-3 | 1,257,560 | 746 | 0.92 (0.83-1.02) |  |
| *p* for trend |  |  | 0.162 |  |
| **TC (mmol/L)** | 3,937,755 | 2,463 | 1.06 (1.02-1.10) | 0.004 |
| 2-4.70 | 1,251,879 | 612 | Reference |  |
| 4.71-5.70 | 1,365,965 | 846 | 1.10 (0.99-1.21) |  |
| 5.71-9 | 1,319,911 | 1,005 | 1.17 (1.06-1.29) |  |
| *p* for trend |  |  | 0.002 |  |
| **TG (log_2_ mmol/L)** | 3,261,922 | 2,111 | 1.00 (0.96-1.05) | 0.819 |
| 0.50-1.09 | 1,105,604 | 656 | Reference |  |
| 1.10-1.70 | 1,142,259 | 771 | 0.98 (0.89-1.10) |  |
| 1.71-6 | 1,014,059 | 684 | 1.03 (0.92-1.15) |  |
| *p* for trend |  |  | 0.614 |  |

HR, hazard ratios; CI, confidence interval; TC, total cholesterol; HDL-c, high-density lipoprotein cholesterol; LDL-c, low-density lipoprotein cholesterol; TC, total cholesterol, TG, triglycerides, HbA1c, haemoglobin A1c.

^1^ Adjusted for age (as the time scale of the model), sex, Townsend deprivation index, and use of lipid-lowering medications (as a time-varying covariate).

**eTable 7**. Association (per 1-SD increase) of the first measurement of lipid blood biomarkers with the incidence of amyotrophic lateral sclerosis stratified by sex, median age, and lipid-lowering treatment.

| **Biomarker** | **N** | **No of events** | **Adjusted^1^**  **HR (95%CI)** | **P_interaction_** |
| --- | --- | --- | --- | --- |
| **LDL-c (mmol/L)** |  |  |  |  |
| Males | 1,460,259 | 1,081 | 1.08 (1.02-1.15) | 0.395 |
| Females | 1,560,327 | 810 | 1.04 (0.97-1.11) |  |
| **HDL-c (mmol/L)** |  |  |  |  |
| Males | 1,808,304 | 1,275 | 0.97 (0.91-1.03) | 0.316 |
| Females | 1,931,222 | 967 | 1.02 (0.96-1.08) |  |
| **TC (mmol/L)** |  |  |  |  |
| Males | 1,902,439 | 1,410 | 1.07 (1.02-1.13) | 0.544 |
| Females | 2,035,316 | 1,053 | 1.04 (0.98-1.10) |  |
| **TG (log_2_ mmol/L)** |  |  |  |  |
| Males | 1,583,848 | 1,197 | 1.03 (0.97-1.09) | 0.278 |
| Females | 1,678,074 | 914 | 0.97 (0.91-1.04) |  |
| **LDL-c (mmol/L)** |  |  |  |  |
| <median age | 1,503,919 | 443 | 1.07 (0.97-1.18) | 0.057 |
| ≥median age | 1,516,667 | 1,448 | 1.06 (1.01-1.11) |  |
| **HDL-c (mmol/L)** |  |  |  |  |
| <median age | 1,919,639 | 543 | 0.92 (0.83-1.01) | 0.841 |
| ≥median age | 1,819,887 | 1,699 | 1.02 (0.97-1.07) |  |
| **TC (mmol/L)** |  |  |  |  |
| <median age | 1,962,053 | 593 | 0.96 (0.88-1.05) | 0.003 |
| ≥median age | 1,975,702 | 1,870 | 1.08 (1.04-1.13) |  |
| **TG (log_2_ mmol/L)** |  |  |  |  |
| <median age | 1,639,396 | 521 | 1.05 (0.96-1.15) | 0.452 |
| ≥median age | 1,622,526 | 1,590 | 0.99 (0.94-1.04) |  |
| **LDL-c (mmol/L)** |  |  |  |  |
| LLT users | 1,109,622 | 717 | 1.04 (0.97-1.11) | 0.193 |
| LLT non-users | 1,910,964 | 1,174 | 1.09 (1.03-1.16) |  |
| **HDL-c (mmol/L)** |  |  |  |  |
| LLT users | 1,303,481 | 840 | 1.01 (0.94-1.10) | 0.373 |
| LLT non-users | 2,436,045 | 1,402 | 0.98 (0.93-1.03) |  |
| **TC (mmol/L)** |  |  |  |  |
| LLT users | 1,333,095 | 881 | 1.05 (0.99-1.12) | 0.491 |
| LLT non-users | 2,604,660 | 1,582 | 1.07 (1.02-1.13) |  |
| **TG (log_2_ mmol/L)** |  |  |  |  |
| LLT users | 1,188,738 | 803 | 1.03 (0.95-1.11) | 0.690 |
| LLT non-users | 2,073,184 | 1,308 | 1.00 (0.94-1.07) |  |

HR, hazard ratios; CI, confidence interval; TC, total cholesterol; HDL-c, high-density lipoprotein cholesterol; LDL-c, low-density lipoprotein cholesterol; TC, total cholesterol, TG, triglycerides, HbA1c, haemoglobin A1c.

^1^ Adjusted for age (as the time scale of the model), Townsend deprivation index, and use of lipid-lowering medications (as a time-varying covariate).

**eTable 8**. Association of the first measurement of lipid blood biomarkers with the incidence of frontotemporal dementia. For each biomarker, the first row represents its association modelled continuously (per 1-SD increase), while subsequent rows evaluate the association categorically across tertiles, with the first tertile serving as the reference group.

| **Biomarker** | **N** | **No of events** | **Adjusted^1^**  **HR (95%CI)** | **P-value** |
| --- | --- | --- | --- | --- |
| **LDL-c (mmol/L)** | 3,021,140 | 570 | 1.02 (0.94-1.11) | 0.615 |
| 1-2.60 | 932,958 | 158 | Reference |  |
| 2.61-3.50 | 1,056,015 | 189 | 1.04 (0.84-1.29) |  |
| 3.51-10 | 1,032,167 | 223 | 1.10 (0.90-1.35) |  |
| *p* for trend |  |  | 0.432 |  |
| **HDL-c (mmol/L)** | 3,740,197 | 675 | 1.01 (0.93-1.09) | 0.794 |
| 0.50-1.20 | 1,301,045 | 230 | Reference |  |
| 1.21-1.56 | 1,181,321 | 214 | 0.97 (0.80-1.17) |  |
| 1.57-3 | 1,257,831 | 231 | 0.96 (0.80-1.15) |  |
| *p* for trend |  |  | 0.657 |  |
| **TC (mmol/L)** | 3,938,478 | 723 | 1.08 (1.01-1.16) | 0.031 |
| 2-4.70 | 1,252,002 | 188 | Reference |  |
| 4.71-5.70 | 1,366,304 | 240 | 1.03 (0.84-1.26) |  |
| 5.71-9 | 1,320,172 | 295 | 1.13 (0.93-1.36) |  |
| *p* for trend |  |  | 0.192 |  |
| **TG (log_2_ mmol/L)** | 3,262,536 | 621 | 1.01 (0.93-1.10) | 0.817 |
| 0.50-1.09 | 1,105,810 | 203 | Reference |  |
| 1.10-1.70 | 1,142,478 | 205 | 0.83 (0.68-1.02) |  |
| 1.71-6 | 1,014,248 | 213 | 1.01 (0.83-1.24) |  |
| *p* for trend |  |  | 0.890 |  |

HR, hazard ratios; CI, confidence interval; TC, total cholesterol; HDL-c, high-density lipoprotein cholesterol; LDL-c, low-density lipoprotein cholesterol; TC, total cholesterol, TG, triglycerides, HbA1c, haemoglobin A1c.

^1^ Adjusted for age (as the time scale of the model), sex, Townsend deprivation index, and use of lipid-lowering medications (as a time-varying covariate).

**eTable 9**. Association (per 1-SD increase) of the first measurement of lipid blood biomarkers with the incidence of frontotemporal dementia stratified by sex, median age, and lipid-lowering treatment.

| **Biomarker** | **N** | **No of events** | **Adjusted^1^**  **HR (95%CI)** | **P_interaction_** |
| --- | --- | --- | --- | --- |
| **LDL-c (mmol/L)** |  |  |  |  |
| Males | 1,460,621 | 318 | 0.99 (0.89-1.11) | 0.481 |
| Females | 1,560,519 | 252 | 1.06 (0.93-1.19) |  |
| **HDL-c (mmol/L)** |  |  |  |  |
| Males | 1,808,763 | 379 | 1.05 (0.94-1.18) | 0.306 |
| Females | 1,931,434 | 296 | 0.97 (0.87-1.09) |  |
| **TC (mmol/L)** |  |  |  |  |
| Males | 1,902,925 | 406 | 1.07 (0.97-1.19) | 0.729 |
| Females | 2,035,553 | 317 | 1.10 (0.98-1.22) |  |
| **TG (log_2_ mmol/L)** |  |  |  |  |
| Males | 1,584,259 | 345 | 1.00 (0.90-1.12) | 0.884 |
| Females | 1,678,277 | 276 | 1.02 (0.89-1.17) |  |
| **LDL-c (mmol/L)** |  |  |  |  |
| <median age | 1,504,092 | 136 | 0.95 (0.80-1.14) | 0.398 |
| ≥median age | 1,517,048 | 434 | 1.03 (0.94-1.13) |  |
| **HDL-c (mmol/L)** |  |  |  |  |
| <median age | 1,919,853 | 179 | 0.91 (0.76-1.08) | 0.145 |
| ≥median age | 1,820,344 | 496 | 1.06 (0.97-1.15) |  |
| **TC (mmol/L)** |  |  |  |  |
| <median age | 1,962,254 | 182 | 1.01 (0.86-1.19) | 0.205 |
| ≥median age | 1,976,224 | 541 | 1.10 (1.01-1.19) |  |
| **TG (log_2_ mmol/L)** |  |  |  |  |
| <median age | 1,639,575 | 164 | 1.03 (0.89-1.19) | 0.763 |
| ≥median age | 1,622,961 | 457 | 1.00 (0.90-1.11) |  |
| **LDL-c (mmol/L)** |  |  |  |  |
| LLT users | 1,109,823 | 231 | 1.01 (0.90-1.15) | 0.143 |
| LLT non-users | 1,911,317 | 339 | 1.03 (0.92-1.16) |  |
| **HDL-c (mmol/L)** |  |  |  |  |
| LLT users | 1,303,713 | 263 | 1.07 (0.94-1.22) | 0.631 |
| LLT non-users | 2,436,484 | 412 | 0.97 (0.87-1.08) |  |
| **TC (mmol/L)** |  |  |  |  |
| LLT users | 1,333,310 | 268 | 1.11 (1.00-1.24) | 0.878 |
| LLT non-users | 2,605,168 | 455 | 1.07 (0.76-1.18) |  |
| **TG (log_2_ mmol/L)** |  |  |  |  |
| LLT users | 1,188,961 | 246 | 1.03 (0.91-1.18) | 0.906 |
| LLT non-users | 2,073,575 | 375 | 1.00 (0.89-1.23) |  |

HR, hazard ratios; CI, confidence interval; TC, total cholesterol; HDL-c, high-density lipoprotein cholesterol; LDL-c, low-density lipoprotein cholesterol; TC, total cholesterol, TG, triglycerides, HbA1c, haemoglobin A1c.

^1^ Adjusted for age (as the time scale of the model), Townsend deprivation index, and use of lipid-lowering medications (as a time-varying covariate).


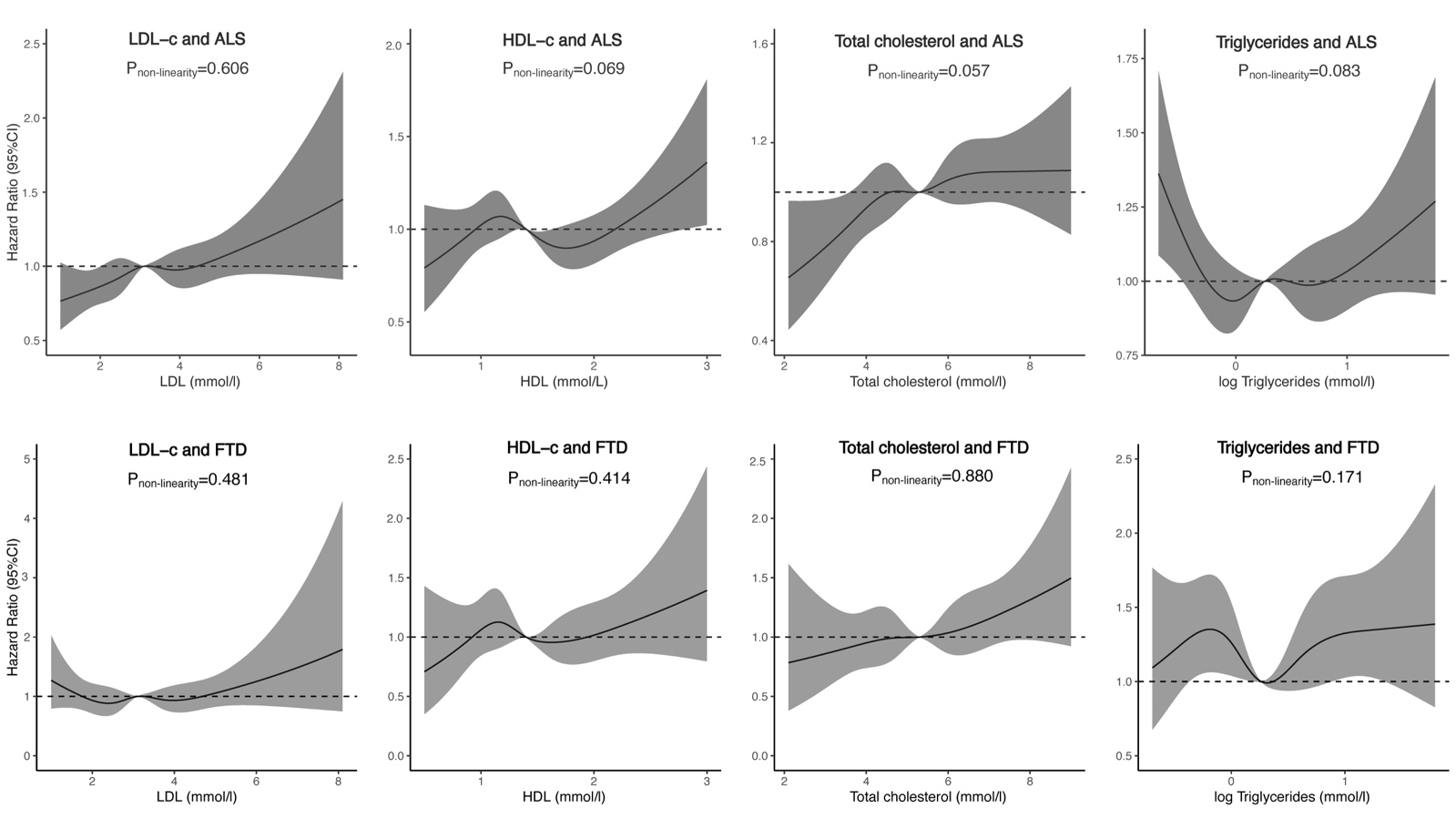


**eFigure 1**. Non-linear relationship between the lipid blood biomarkers and amyotrophic lateral sclerosis (ALS) using the Cox proportional models with restricted cubic splines and age at sampling as the time scale. Knots were placed at the 5th, 50th, and 95th percentile, with the 50th (median) as the reference. LDL-c, Low-Density Lipoprotein cholesterol; HDL-c, High-Density Lipoprotein cholesterol; TC, Total Cholesterol; TG, Triglycerides.

**eTable 10.** Association of the first measurement of lipid blood biomarkers with the incidence of amyotrophic lateral sclerosis after excluding those with a history of FTD diagnosis. For each biomarker, the first row represents its association modelled continuously (per 1-SD increase), while subsequent rows evaluate the association categorically across tertiles, with the first tertile serving as the reference group.

| **Biomarker** | **N** | **No of events** | **Adjusted^1^**  **HR (95%CI)** | **P-value** |
| --- | --- | --- | --- | --- |
| **LDL-c (mmol/L)** | 3,019,859 | 1,878 | 1.06 (1.02-1.11) | 0.003 |
| 1-2.60 | 932,583 | 487 | Reference |  |
| 2.61-3.50 | 1,055,596 | 643 | 1.11 (0.99-1.25) |  |
| 3.51-10 | 1,031,680 | 748 | 1.16 (1.04-1.30) |  |
| *p* for trend |  |  | 0.008 |  |
| **HDL-c (mmol/L)** | 3,738,656 | 2,225 | 0.99 (0.95-1.04) | 0.833 |
| 0.50-1.20 | 1,300,557 | 758 | Reference |  |
| 1.21-1.56 | 1,180,834 | 728 | 0.99 (0.89-1.10) |  |
| 1.57-3 | 1,257,265 | 739 | 0.92 (0.83-1.02) |  |
| *p* for trend |  |  | 0.123 |  |
| **TC (mmol/L)** | 3,936,839 | 2,443 | 1.06 (1.02-1.10) | 0.003 |
| 2-4.70 | 1,251,615 | 606 | Reference |  |
| 4.71-5.70 | 1,365,661 | 839 | 1.10 (0.99-1.22) |  |
| 5.71-9 | 1,319,563 | 999 | 1.17 (1.06-1.30) |  |
| *p* for trend |  |  | 0.001 |  |
| **TG (log_2_ mmol/L)** | 3,261,143 | 2,096 | 1.01 (0.96-1.05) | 0.753 |
| 0.50-1.09 | 1,105,349 | 649 | Reference |  |
| 1.10-1.70 | 1,141,993 | 767 | 0.99 (0.89-1.10) |  |
| 1.71-6 | 1,013,801 | 680 | 1.03 (0.92-1.16) |  |
| *p* for trend |  |  | 0.549 |  |

HR, hazard ratios; CI, confidence interval; TC, total cholesterol; HDL-c, high-density lipoprotein cholesterol; LDL-c, low-density lipoprotein cholesterol; TC, total cholesterol, TG, triglycerides, HbA1c, haemoglobin A1c.

1 Adjusted for age (as the time scale of the model), sex, Townsend deprivation index, and use of lipid-lowering medications (as a time-varying covariate).

**eTable 11**. Association of the first measurement of lipid blood biomarkers with the incidence of frontotemporal dementia after excluding those with a history of ALS diagnosis. For each biomarker, the first row represents its association modelled continuously (per 1-SD increase), while subsequent rows evaluate the association categorically across tertiles, with the first tertile serving as the reference group.

| **Biomarker** | **N** | **No of events** | **Adjusted^1^**  **HR (95%CI)** | **P-value** |
| --- | --- | --- | --- | --- |
| **LDL-c (mmol/L)** | 3,018,538 | 555 | 1.02 (0.94-1.11) | 0.564 |
| 1-2.60 | 932,250 | 154 | Reference |  |
| 2.61-3.50 | 1,055,135 | 185 | 1.04 (0.84-1.30) |  |
| 3.51-10 | 1,031,152 | 216 | 1.10 (0.90-1.35) |  |
| *p* for trend |  |  | 0.372 |  |
| **HDL-c (mmol/L)** | 3,737,077 | 656 | 1.01 (0.93-1.09) | 0.891 |
| 0.50-1.20 | 1,299,985 | 221 | Reference |  |
| 1.21-1.56 | 1,180,305 | 212 | 0.99 (0.82-1.20) |  |
| 1.57-3 | 1,256,787 | 223 | 0.96 (0.79-1.16) |  |
| *p* for trend |  |  | 0.656 |  |
| **TC (mmol/L)** | 3,935,092 | 705 | 1.09 (1.01-1.17) | 0.026 |
| 2-4.70 | 1,251,113 | 184 | Reference |  |
| 4.71-5.70 | 1,365,125 | 233 | 1.02 (0.84-1.25) |  |
| 5.71-9 | 1,318,154 | 288 | 1.12 (0.93-1.36) |  |
| *p* for trend |  |  | 0.201 |  |
| **TG (log_2_ mmol/L)** | 3,259,651 | 604 | 1.01 (0.93-1.10) | 0.806 |
| 0.50-1.09 | 1,104,915 | 196 | Reference |  |
| 1.10-1.70 | 1,141,430 | 201 | 0.85 (0.69-1.04) |  |
| 1.71-6 | 1,013,306 | 207 | 1.02 (0.84-1.25) |  |
| *p* for trend |  |  | 0.800 |  |

HR, hazard ratios; CI, confidence interval; TC, total cholesterol; HDL-c, high-density lipoprotein cholesterol; LDL-c, low-density lipoprotein cholesterol; TC, total cholesterol, TG, triglycerides, HbA1c, haemoglobin A1c.

^1^ Adjusted for age (as the time scale of the model), sex, Townsend deprivation index, and use of lipid-lowering medications (as a time-varying covariate).

**eTable 12**. Association of the first measurement of LDL-c (considering additionally greater levels than 10 mmol/L) with the incidence of amyotrophic lateral (ALS) sclerosis and frontotemporal dementia (FTD). The first row represents its continuous scale measurement (per 1-SD increase), and subsequent rows categorise LDL-c into tertiles, with the first tertile used as the reference category.

|  | **N** | **No of events** | **Adjusted^1^**  **HR (95%CI)** | **P-value** |
| --- | --- | --- | --- | --- |
| **Amyotrophic lateral sclerosis** | | |  |  |
| **LDL-c (mmol/L)** | 3,020,778 | 1,891 | 1.06 (1.01-1.10) | 0.001 |
| 1-2.60 | 932,819 | 491 | Reference |  |
| 2.61-3.50 | 1,055,829 | 646 | 1.11 (0.99-1.24) |  |
| 3.50-59 | 1,032,130 | 754 | 1.16 (1.04-1.30) |  |
| *p* for trend |  |  | 0.008 |  |
| **Frontotemporal dementia** | |  |  |  |
| **LDL-c (mmol/L)** | 3,021,327 | 570 | 1.02 (0.94-1.10) | 0.622 |
| 1-2.60 | 932,984 | 159 | Reference |  |
| 2.61-3.50 | 1,055,989 | 189 | 1.04 (0.84-1.29) |  |
| 3.50-59 | 1,032,354 | 223 | 1.10 (0.90-1.34) |  |
| *p* for trend |  |  | 0.346 |  |

HR, hazard ratios; CI, confidence interval; LDL-c, low-density lipoprotein cholesterol.

^1^ Adjusted for age (as the time scale of the model), sex, Townsend deprivation index, and use of lipid-lowering medications (as a time-varying covariate).

**eTable 13**. Association of the first measurement of lipid blood biomarkers with the incidence of amyotrophic lateral sclerosis adjusting for further covariates. For each biomarker, the first row represents its association modelled continuously (per 1-SD increase), while subsequent rows evaluate the association categorically across tertiles, with the first tertile serving as the reference group.

| **Biomarker** | **N** | **No of events** | **Adjusted^1^**  **HR (95%CI)** | **P-value** |
| --- | --- | --- | --- | --- |
| **LDL-c (mmol/L)** | 3,020,586 | 1,891 | 1.06 (1.01-1.11) | 0.015 |
| 1-2.60 | 932,796 | 491 | Reference |  |
| 2.61-3.50 | 1,055,833 | 646 | 1.10 (0.97-1.24) |  |
| 3.51-10 | 1,031,957 | 754 | 1.15 (1.02-1.29) |  |
| *p* for trend |  |  | 0.026 |  |
| **HDL-c (mmol/L)** | 3,739,526 | 2,242 | 0.99 (0.95-1.05) | 0.993 |
| 0.50-1.20 | 1,300,860 | 768 | Reference |  |
| 1.21-1.56 | 1,181,106 | 728 | 0.98 (0.88-1.08) |  |
| 1.57-3 | 1,257,560 | 746 | 0.92 (0.82-1.03) |  |
| *p* for trend |  |  | 0.155 |  |
| **TC (mmol/L)** | 3,937,755 | 2,463 | 1.05 (1.01-1.09) | 0.024 |
| 2-4.70 | 1,251,879 | 612 | Reference |  |
| 4.71-5.70 | 1,365,965 | 846 | 1.08 (0.97-1.20) |  |
| 5.71-9 | 1,319,911 | 1,005 | 1.15 (1.03-1.28) |  |
| *p* for trend |  |  | 0.01 |  |
| **TG (log_2_ mmol/L)** | 3,261,922 | 2,111 | 1.00 (0.95-1.05) | 0.906 |
| 0.50-1.09 | 1,105,604 | 656 | Reference |  |
| 1.10-1.70 | 1,142,259 | 771 | 0.98 (0.89-1.09) |  |
| 1.71-6 | 1,014,059 | 684 | 1.01 (0.90-1.13) |  |
| *p* for trend |  |  | 0.846 |  |

HR, hazard ratios; CI, confidence interval; TC, total cholesterol; HDL-c, high-density lipoprotein cholesterol; LDL-c, low-density lipoprotein cholesterol; TC, total cholesterol, TG, triglycerides, HbA1c, haemoglobin A1c.

^1^ Adjusted for age (as the time scale of the model), sex, Townsend deprivation index, and use of lipid-lowering medications (as a time-varying covariate), smoking, BMI, comorbidities (cardiovascular diseases, peripheral vascular diseases, atrial fibrillation, diabetes, chronic kidney disease).

**eTable 14**. Association of the first measurement of lipid blood biomarkers with the incidence of frontotemporal dementia adjusting for further covariates. For each biomarker, the first row represents its association modelled continuously (per 1-SD increase), while subsequent rows evaluate the association categorically across tertiles, with the first tertile serving as the reference group.

| **Biomarker** | **N** | **No of events** | **Adjusted^1^**  **HR (95%CI)** | **P-value** |
| --- | --- | --- | --- | --- |
| **LDL-c (mmol/L)** | 3,021,140 | 570 | 1.00 (0.92-1.09) | 0.994 |
| 1-2.60 | 932,958 | 158 | Reference |  |
| 2.61-3.50 | 1,056,015 | 189 | 1.00 (0.81-1.24) |  |
| 3.51-10 | 1,032,167 | 223 | 1.05 (0.85-1.31) |  |
| *p* for trend |  |  | 0.601 |  |
| **HDL-c (mmol/L)** | 3,740,197 | 675 | 0.99 (0.91-1.09) | 0.903 |
| 0.50-1.20 | 1,301,045 | 230 | Reference |  |
| 1.21-1.56 | 1,181,321 | 214 | 0.96 (0.79-1.16) |  |
| 1.57-3 | 1,257,831 | 231 | 0.92 (0.75-1.14) |  |
| *p* for trend |  |  | 0.460 |  |
| **TC (mmol/L)** | 3,938,478 | 723 | 1.06 (0.98-1.14) | 0.114 |
| 2-4.70 | 1,252,002 | 188 | Reference |  |
| 4.71-5.70 | 1,366,304 | 240 | 0.99 (0.81-1.20) |  |
| 5.71-9 | 1,320,172 | 295 | 1.07 (0.88-1.30) |  |
| *p* for trend |  |  | 0.439 |  |
| **TG (log_2_ mmol/L)** | 3,262,536 | 621 | 1.00 (0.92-1.09) | 0.989 |
| 0.50-1.09 | 1,105,810 | 203 | Reference |  |
| 1.10-1.70 | 1,142,478 | 205 | 0.83 (0.68-1.02) |  |
| 1.71-6 | 1,014,248 | 213 | 0.99 (0.80-1.22) |  |
| *p* for trend |  |  | 0.979 |  |

HR, hazard ratios; CI, confidence interval; TC, total cholesterol; HDL-c, high-density lipoprotein cholesterol; LDL-c, low-density lipoprotein cholesterol; TC, total cholesterol, TG, triglycerides, HbA1c, haemoglobin A1c.

^1^ Adjusted for age (as the time scale of the model), sex, Townsend deprivation index, and use of lipid-lowering medications (as a time-varying covariate only in lipid cohorts), smoking, BMI, comorbidities (cardiovascular diseases, peripheral vascular diseases, atrial fibrillation, diabetes, chronic kidney disease).

**eTable 15**. Baseline characteristics of people with at least one creatine kinase measurement with and without ALS/ with and without FTD at least four years after the first creatine kinase measurement.

|  | **People without ALS (n=496,383)** | **People with ALS (n=438)** | **People without FTD (n=496,884)** | **People with FTD (n=142)** |
| --- | --- | --- | --- | --- |
| **Follow-up time, median (IQR), yrs.** | 10.2 (6.7-14.6) | 8.8 (6.2-12.7) | 10.2 (6.7-14.6) | 9.7 (6.8-12.7) |
| **Creatine kinase, IU/L** |  |  |  |  |
| Median (IQR) | 97 (69-143) | 105 (72-155) | 97 (69-143) | 101.5 (74-148) |
| Mean (SD) | 121.5 (87.3) | 130.3 (88.2) | 121.5 (87.3) | 121.8 (10.3) |
| **Age at sampling, median (IQR), yrs.** | 62.1 (51.4-71.6) | 54.4 (58.1-71.2) | 62.1 (51.4-71.6) | 65.7 (50.2-70.1) |
| **Sex, males** | 236,614 (47.7) | 247 (56.4) | 236,905 (47.7) | 83 (58.5) |
| **Townsend deprivation index, quintiles** |  |  |  |  |
| Least deprived | 158,692 (32) | 178 (40.6) | 158,912 (32) | 49 (34.5) |
| - | 122,153 (24.6) | 127 (29) | 122,291 (24.6) | 41 (28.9) |
| - | 93,046 (18.7) | 54 (12.3) | 93,112 (18.7) | 21 (17.8) |
| - | 69,421 (14) | 46 (10.5) | 69,461 (14) | 22 (15.5) |
| Most deprived | 51,446 (10.4) | 32 (7.3) | 51,482 (10.4) | 8 (5.6) |
| Missing | 1,625 (0.3) | <5 | 1,626 (0.3) | <5 |
| **Body mass index, mean (SD),** $\frac{\boldsymbol{kg}}{\boldsymbol{m}^{\boldsymbol{2}}}$ | 28.4 (5.3) | 27.8 (4.9) | 28.4 (5.3) | 27.7 (4.7) |
| Missing | 155,446 (31.3) | 141 (32.2) | 155,619 (31.3) | 37 (26) |
| **Smoking status** |  |  |  |  |
| Non-smokers | 182,839 (36.8) | 162 (37) | 182,028 (36.8) | 41 (28.9) |
| Ex-smokers | 143,291 (28.9) | 129 (29.5) | 143,434 (28.9) | 48 (33.8) |
| Current smokers | 82,629 (16.7) | 60 (13.7) | 82,690 (16.7) | 28 (19.7) |
| Missing | 87,624 (17.6) | 87 (19.8) | 87,732 (17.6) | 25 (17.6) |
| **Comorbidity (any before the index date)** |  |  |  |  |
| Diabetes | 72,335 (14.6) | 54 (12.3) | 72,396 (14.6) | 23 (16.2) |
| Cardiovascular diseases | 76,312 (15.4) | 86 (19.6) | 76,420 (15.4) | 20 (14.1) |
| Peripheral vascular disease | 7,716 (1.6) | <5 | 7,720 (1.6) | <5 |
| Chronic kidney disease | 27,741 (5.6) | 19 (4.3) | 27,768 (5.6) | <5 |
| Atrial fibrillation | 17,877 (3.6) | 12 (2.7) | 17,877 (3.6) | <5 |
| Family history of MND | 17 (0.003) | <5 | 17 (0.003) | <5 |
| Family history of angina | 1,246 (0.3) | <5 | 1,246 (0.3) | <5 |

ALS, Amyotrophic Lateral Sclerosis; FTD, Frontotemporal dementia; MND, Motor Neuron Disease

All figures are presented as absolute numbers (percentage) unless otherwise specified.

Counts less than 5 are suppressed for confidentiality.

**eTable 16**. Baseline characteristics of people with at least one creatinine measurement with and without ALS/ with and without FTD at least four years after the first creatinine measurement.

|  | **People without ALS (n=4,617,660)** | **People with ALS (n=2,695)** | **People without FTD (n=4,620,427)** | **People with FTD (n=781)** |
| --- | --- | --- | --- | --- |
| **Follow-up time, median (IQR), yrs.** | 9.3 (6.2-14.9) | 9.4 (6.4-13.4) | 9.3 (6.2-14.9) | 10.5 (6.7-14.6) |
| **Creatinine, umol/L** |  |  |  |  |
| Median (IQR) | 80 (68-92) | 86 (75-97) | 80 (68-92) | 85 (73-97) |
| Mean (SD) | 81.1 (17.6) | 86.7 (17) | 81.1 (17.6) | 86 (16.9) |
| **Age at sampling, median (IQR), yrs.** | 52.7 (41.1-65.2) | 62.3 (54.7-69.2) | 52.7 (41.1-65.2) | 62.9 (54.7-69.7) |
| **Sex, males** | 2,118,265 (45.9) | 1,497 (55.6) | 2,118,895 (45.9) | 416 (53.3) |
| **Townsend deprivation index** |  |  |  |  |
| Least deprived | 1,346,033 (29.2) | 996 (37) | 1,347,078 (29.2) | 249 (31.9) |
| - | 1,083,597 (23.5) | 706 (26.2) | 1,084,302 (23.5) | 190 (24.3) |
| - | 878,773 (19) | 418 (15.5) | 879,209 (19) | 145 (15.6) |
| - | 707,689 (15.3) | 318 (11.8) | 708,022 (15.3) | 105 (13.4) |
| Most deprived | 580,116 (12.6) | 249 (9.2) | 580,335 (12.6) | 90 (11.5) |
| Missing | 21,452 (0.5) | 8 (0.3) | 21,461 (0.5) | <5 |
| **Body mass index, mean (SD),** $\frac{\boldsymbol{kg}}{\boldsymbol{m}^{\boldsymbol{2}}}$ | 27.7 (5.5) | 27.6 (5) | 27.7 (5.5) | 27.5 (4.7) |
| Missing | 2,810,763 (60.1) | 1,597 (59.2) | 2,812,436 (60.9) | 447 (57.2) |
| **Smoking status** |  |  |  |  |
| Non-smokers | 1,244,056 (26.9) | 676 (25.1) | 1,244,734 (26.9) | 199 (25.5) |
| Ex-smokers | 667,770 (14.5) | 443 (16.4) | 668,237 (14.5) | 122 (15.6) |
| Current smokers | 633,905 (13.7) | 337 (12.5) | 634,231 (13.7) | 106 (13.6) |
| Missing | 2,071,929 (44.9) | 1,239 (46) | 2,073,255 (44.9) | 354 (45.3) |
| **Comorbidity (any before the index date)** |  |  |  |  |
| Diabetes | 200,669 (4.4) | 142 (5.3) | 200,814 (4.4) | 42 (5.4) |
| Cardiovascular diseases | 134,026 (2.9) | 145 (5.4) | 134,187 (2.9) | 37 (4.7) |
| Peripheral vascular disease | 12,680 (0.3) | 12 (0.5) | 12,688 (0.3) | <5 |
| Chronic kidney disease | 15,607 (0.3) | 12 (0.5) | 15,621 (0.3) | <5 |
| Atrial fibrillation | 38,912 (0.8) | 21 (0.8) | 38,941 (0.8) | 8 (1) |
| Family history of MND | 78 (0.001) | <5 | 78 (0.001) | <5 |
| Family history of angina | 4,226 (0.1) | <5 | 4,227 (0.1) | <5 |

ALS, Amyotrophic Lateral Sclerosis; FTD, Frontotemporal dementia; MND, Motor Neuron Disease

All figures are presented as absolute numbers (percentage) unless otherwise specified.

Counts less than 5 are suppressed for confidentiality.

**eTable 17**. Baseline characteristics of people with at least one HbA1c measurement with and without ALS/ with and without FTD at least four years after the first HbA1c measurement.

|  | **People without ALS (n=2,901,464)** | **People with ALS (n=989)** | **People without FTD (n=2,902,583)** | **People with FTD (n=368)** |
| --- | --- | --- | --- | --- |
| **Follow-up time, median (IQR), yrs.** | 6.8 (5.3-8.8) | 6.5 (5.1-9.3) | 6.8 (5.3-8.8) | 7 (5.2-9.7) |
| **HbA1c, mmol/L** |  |  |  |  |
| Median (IQR) | 38 (35-43) | 40 (37-48.6) | 38 (35-43) |  |
| Mean (SD) | 41.9 (12.9) | 45.7 (15.2) | 41.9 (12.9) |  |
| **Age at sampling, median (IQR), yrs.** | 57.3 (46.3-68.7) | 65.4 (57.1-72.1) | 57.3 (46.3-68.7) | 67 (58.5-73) |
| **Sex, males** | 1,353,307 (46.6) | 690 (58.7) | 1,354,031 (46.6) | 307 (56.3) |
| **Townsend deprivation index** |  |  |  |  |
| Least deprived | 822,797 (28.4) | 344 (34.8) | 823,242 (28.4) | 103 (28) |
| - | 656,423 (22.7) | 211 (21.3) | 659,686 (22.7) | 87 (23.6) |
| - | 549,558 (18.9) | 176 (17.8) | 549,718 (18.9) | 67 (18.2) |
| - | 457,540 (15.8) | 138 (14) | 457,689 (15.8) | 56 (15.2) |
| Most deprived | 397,830 (13.7) | 115 (11.6) | 397,925 (13.7) | 53 (14.4) |
| Missing | 14,316 (0.5) | 5 (0.5) | 14,323 (0.5) | <5 |
| **Body mass index, mean (SD),** $\frac{\boldsymbol{kg}}{\boldsymbol{m}^{\boldsymbol{2}}}$ | 28.5 (5.7) | 28.5 (5.2) | 28.5 (5.7) | 28.3 (5.1) |
| Missing | 1,077,530 (37.1) | 295 (29.8) | 1,077,890 (37.1) | 119 (32.3) |
| **Smoking status** |  |  |  |  |
| Non-smokers | 1,006,904 (34.7) | 334 (33.7) | 1,007,275 (34.7) | 110 (29.9) |
| Ex-smokers | 670,540 (23.4) | 262 (26.5) | 678,862 (23.4) | 112 (30.4) |
| Current smokers | 451,777 (15.6) | 149 (15.1) | 451,930 (15.6) | 60 (16.3) |
| Missing | 764,243 (26.3) | 244 (24.7) | 764,516 (26.3) | 86 (23.4) |
| **Comorbidity (any before the index date)** |  |  |  |  |
| Diabetes | 292,200 (10.1) | 215 (21.7) | 292,387 (10.1) | 67 (18.2) |
| Cardiovascular diseases | 181,434 (6.3) | 105 (10.6) | 181,595 (6.3) | 36 (9.8) |
| Peripheral vascular disease | 19,221 (0.7) | 10 (1) | 19,230 (0.7) | 5 (1.4) |
| Chronic kidney disease | 108,829 (3.8) | 52 (5.3) | 108,874 (3.8) | 19 (0.7) |
| Atrial fibrillation | 65,872 (2.3) | 24 (2.4) | 65,906 (2.3) | 7 (1.9) |
| Family history of MND | 122 (0.001) | <5 | 122 (0.001) | <5 |
| Family history of angina | 11,872 (0.4) | <5 | 11,876 (0.4) | <5 |

ALS, Amyotrophic Lateral Sclerosis; FTD, Frontotemporal dementia; MND, Motor Neuron Disease

All figures are presented as absolute numbers (percentage) unless otherwise specified.

Counts less than 5 are suppressed for confidentiality.

**eTable 18**. Association of the first measurement of creatinine, creatine kinase, and HbA1c with the incidence of amyotrophic lateral sclerosis. For each biomarker, the first row represents its association modelled continuously (per 1-SD increase), while subsequent rows evaluate the association categorically across tertiles, with the first tertile serving as the reference group.

| **Biomarker** | **N** | **No of events** | **Adjusted^1^**  **HR (95%CI)** | **P-value** |
| --- | --- | --- | --- | --- |
| **Creatinine (umol/L)** | 4,598,895 | 2,687 | 0.96 (0.92-1.00) | 0.08 |
| 45-71 | 1,452,315 | 503 | Reference |  |
| 71.1-86 | 1,539,497 | 880 | 1.05 (0.94-1.18) |  |
| 86.1-160 | 1,607,083 | 1,304 | 1.01 (0.90-1.14) |  |
| *p* for trend |  |  | 0.964 |  |
| **Creatinine kinase (log_2_ iu/L)** | 495,195 | 437 | 1.12 (1.02-1.24) | 0.017 |
| 25-76 | 159,846 | 122 | Reference |  |
| 76.6-122 | 167,109 | 141 | 1.06 (0.85-1.32) |  |
| 123-800 | 168,240 | 174 | 1.30 (1.04-1.63) |  |
| *p* for trend |  |  | 0.019 |  |
| **HbA1c (log_2_ mmol/L)** | 2,888,132 | 984 | 1.03 (0.95-1.11) | 0.452 |
| 27-36 | 982,506 | 201 | Reference |  |
| 36.1-40 | 873,787 | 294 | 1.07 (0.89-1.29) |  |
| 40.1-126 | 1,031,839 | 489 | 0.96 (0.80-1.16) |  |
| *p* for trend |  |  | 0.586 |  |

HR, hazard ratios; CI, confidence interval; TC, total cholesterol; HDL-c, high-density lipoprotein cholesterol; LDL-c, low-density lipoprotein cholesterol; TC, total cholesterol, TG, triglycerides, HbA1c, haemoglobin A1c.

^1^ Adjusted for age (as the time scale of the model), sex, Townsend deprivation index, and use of anti-diabetic medications (as a time-varying covariate in the HbA1c cohort).

**eTable 19**. Association (per 1-SD increase) of the first measurement of creatinine, creatine kinase, and HbA1c with the incidence of amyotrophic lateral sclerosis stratified by sex and median age.

| **Biomarker** | **N** | **No of events** | **Adjusted^1^**  **HR (95%CI)** | **P_interaction_** |
| --- | --- | --- | --- | --- |
| **Creatinine (umol/L)** |  |  |  |  |
| Males | 2,111,186 | 1,489 | 0.95 (0.89-1.01) | 0.821 |
| Females | 2,478,709 | 1,198 | 0.98 (0.92-1.04) |  |
| **Creatinine kinase (log_2_ iu/L)** |  |  |  |  |
| Males | 236,088 | 242 | 1.20 (1.06-1.36) | 0.129 |
| Females | 258,933 | 190 | 1.01 (0.86-1.20) |  |
| **HbA1c (log_2_ mmol/L)** |  |  |  |  |
| Males | 1,347,776 | 571 | 1.07 (0.97-1.16) | 0.129 |
| Females | 1,539,743 | 406 | 0.96 (0.85-1.09) |  |
| **Creatinine (umol/L)** |  |  |  |  |
| <median age | 2,320,760 | 552 | 0.91 (0.81-1.03) | 0.614 |
| ≥median age | 2,278,135 | 2,135 | 0.96 (0.92-1.01) |  |
| **Creatinine kinase (log_2_ iu/L)** |  |  |  |  |
| <median age | 245,527 | 175 | 1.08 (0.94-1.24) | 0.497 |
| ≥median age | 249,494 | 257 | 1.13 (1.00-1.29) |  |
| **HbA1c (log_2_ mmol/L)** |  |  |  |  |
| <median age | 1,419,642 | 242 | 1.04 (0.91-1.20) | 0.276 |
| ≥median age | 1,467,877 | 735 | 1.01 (0.92-1.10) |  |

HR, hazard ratios; CI, confidence interval; TC, total cholesterol; HDL-c, high-density lipoprotein cholesterol; LDL-c, low-density lipoprotein cholesterol; TC, total cholesterol, TG, triglycerides, HbA1c, haemoglobin A1c.

^1^ Adjusted for age (as the time scale of the model), Townsend deprivation index, and use of anti-diabetic medications (as a time-varying covariate in the HbA1c cohort).

**eTable 20**. Association of the first measurement of creatinine, creatine kinase, and HbA1c with the incidence of frontotemporal dementia. For each biomarker, the first row represents its association modelled continuously (per 1-SD increase), while subsequent rows evaluate the association categorically across tertiles, with the first tertile serving as the reference group.

| **Biomarker** | **N** | **No of events** | **Adjusted^1^**  **HR (95%CI)** | **P-value** |
| --- | --- | --- | --- | --- |
| **Creatinine (umol/L)** | 4,599,745 | 779 | 0.90 (0.83-0.97) | 0.002 |
| 45-71 | 1,452,572 | 161 | Reference |  |
| 71.1-86 | 1,539,731 | 259 | 0.96 (0.78-1.17) |  |
| 86.1-160 | 1,607,442 | 359 | 0.83 (0.67-1.03) |  |
| *p* for trend |  |  | 0.072 |  |
| **Creatinine kinase (log_2_ iu/L)** | 495,399 | 141 | 1.06 (0.90-1.25) | 0.467 |
| 25-76 | 159,876 | 41 | Reference |  |
| 76.6-122 | 167,166 | 45 | 1.00 (0.65-1.55) |  |
| 123-800 | 168,357 | 55 | 1.22 (0.79-1.90) |  |
| *p* for trend |  |  | 0.364 |  |
| **HbA1c (log_2_ mmol/L)** | 2,888,626 | 366 | 0.97 (0.85-1.09) | 0.578 |
| 27-36 | 982,642 | 105 | Reference |  |
| 36.1-40 | 873,962 | 83 | 0.54 (0.40-0.72) |  |
| 40.1-126 | 1,032,022 | 178 | 0.58 (0.44-0.77) |  |
| *p* for trend |  |  | 0.001 |  |

HR, hazard ratios; CI, confidence interval; TC, total cholesterol; HDL-c, high-density lipoprotein cholesterol; LDL-c, low-density lipoprotein cholesterol; TC, total cholesterol, TG, triglycerides, HbA1c, haemoglobin A1c.

^1^ Adjusted for age (as the time scale of the model), sex, Townsend deprivation index, and use of anti-diabetic medications (as a time-varying covariate in the HbA1c cohort).


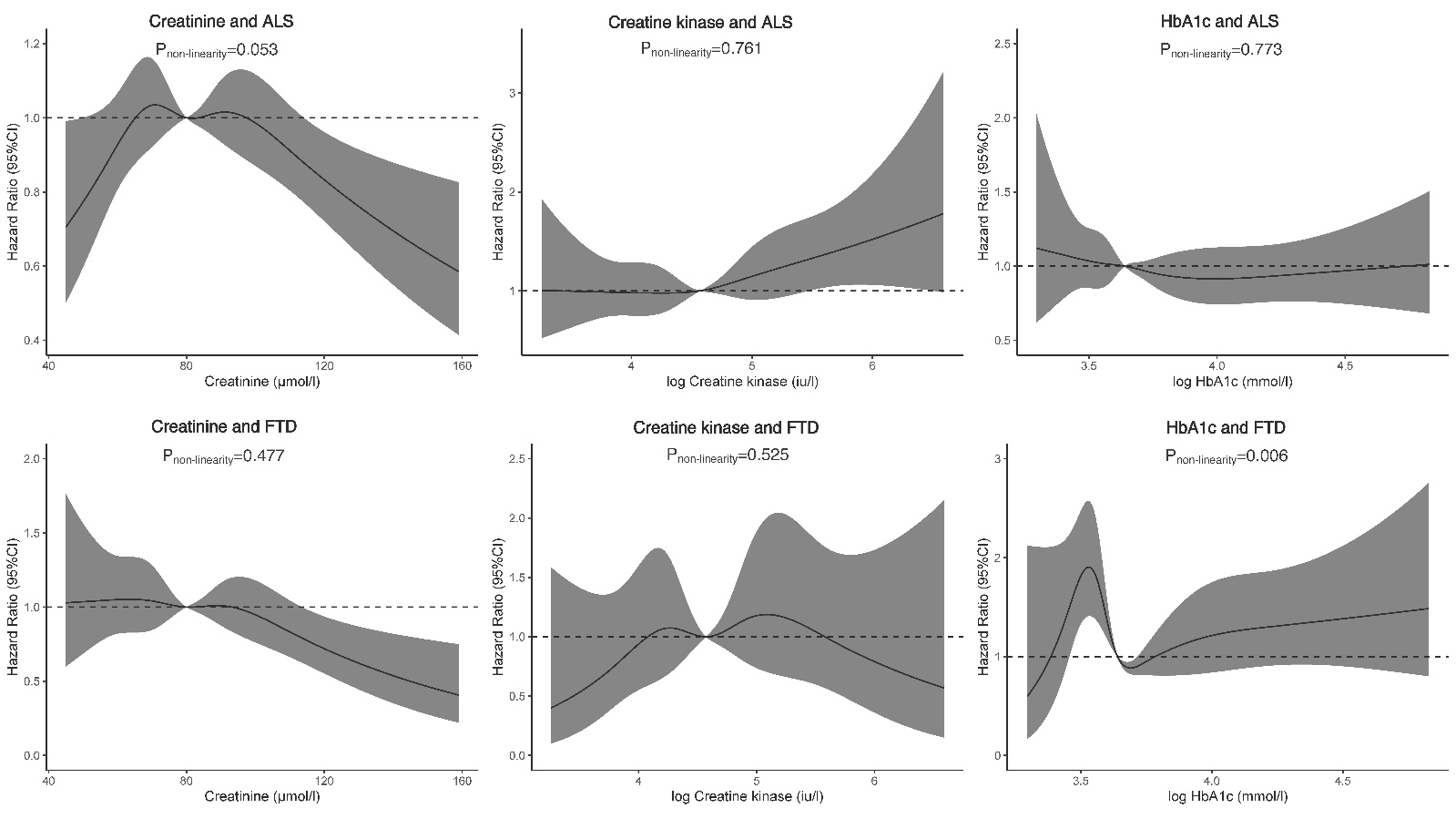


**eFigure 2**. Non-linear relationship between creatinine, creatine kinase, HbA1c and amyotrophic lateral sclerosis (ALS) using the Cox proportional models with restricted cubic splines and age at sampling as the time scale. Knots were placed at the 5th, 50th, and 95th percentile, with the 50th (median) as the reference.

**eTable 21**. Association (per 1-SD increase) of the first measurement of creatinine, creatine kinase, and HbA1c with the incidence of frontotemporal dementia stratified by sex and median age.

| **Biomarker** | **N** | **No of events** | **Adjusted^1^**  **HR (95%CI)** | **P_interaction_** |
| --- | --- | --- | --- | --- |
| **Creatinine (umol/L)** |  |  |  |  |
| Males | 2,111,734 | 414 | 0.90 (0.81-0.99) | 0.847 |
| Females | 2,488,011 | 365 | 0.91 (0.81-1.03) |  |
| **Creatinine kinase (log_2_ iu/L)** |  |  |  |  |
| Males | 236,312 | 82 | 0.99 (0.80-1.23) | 0.244 |
| Females | 259,087 | 59 | 1.18 (0.94-1.48) |  |
| **HbA1c (log_2_ mmol/L)** |  |  |  |  |
| Males | 1,348,455 | 205 | 1.02 (0.86-1.21) | 0.944 |
| Females | 1,540,171 | 161 | 0.89 (0.73-1.08) |  |
| **Creatinine (umol/L)** |  |  |  |  |
| <median age | 2,320,972 | 158 | 0.91 (0.72-1.14) | 0.260 |
| ≥median age | 2,278,773 | 621 | 0.89 (0.82-0.97) |  |
| **Creatinine kinase (log_2_ iu/L)** |  |  |  |  |
| <median age | 245,667 | 43 | 1.21 (0.92-1.58) | 0.400 |
| ≥median age | 249,732 | 98 | 1.00 (0.82-1.23) |  |
| **HbA1c (log_2_ mmol/L)** |  |  |  |  |
| <median age | 1,419,856 | 71 | 0.97 (0.80-1.18) | 0.777 |
| ≥median age | 1,468,770 | 295 | 0.95 (0.82-1.11) |  |

HR, hazard ratios; CI, confidence interval; TC, total cholesterol; HDL-c, high-density lipoprotein cholesterol; LDL-c, low-density lipoprotein cholesterol; TC, total cholesterol, TG, triglycerides, HbA1c, haemoglobin A1c.

^1^ Adjusted for age (as the time scale of the model), Townsend deprivation index, and use of anti-diabetic medications (as a time-varying covariate in the HbA1c cohort).

**eTable 22.** Association of the first measurement of creatinine, creatine kinase, and HbA1c with the incidence of amyotrophic lateral sclerosis after excluding those with a history of FTD diagnosis. For each biomarker, the first row represents its association modelled continuously (per 1-SD increase), while subsequent rows evaluate the association categorically across tertiles, with the first tertile serving as the reference group.

| **Biomarker** | **N** | **No of events** | **Adjusted^1^**  **HR (95%CI)** | **P-value** |
| --- | --- | --- | --- | --- |
| **Creatinine (umol/L)** | 4,597,888 | 2,666 | 0.96 (0.92-1.00) | 0.08 |
| 45-71 | 1,452,085 | 498 | Reference |  |
| 71.1-86 | 1,539,162 | 877 | 1.06 (0.94-1.19) |  |
| 86.1-160 | 1,606,141 | 1,291 | 1.01 (0.90-1.14) |  |
| *p* for trend |  |  | 0.922 |  |
| **Creatinine kinase (log_2_ iu/L)** | 495,021 | 432 | 1.12 (1.02-1.23) | 0.021 |
| 25-76 | 159,790 | 121 | Reference |  |
| 76.6-122 | 167,055 | 139 | 1.06 (0.84-1.32) |  |
| 123-800 | 168,176 | 172 | 1.30 (1.04-1.63) |  |
| *p* for trend |  |  | 0.020 |  |
| **HbA1c (log_2_ mmol/L)** | 2,887,519 | 977 | 1.03 (0.96-1.12) | 0.404 |
| 27-36 | 982,320 | 199 | Reference |  |
| 36.1-40 | 873,634 | 291 | 1.07 (0.89-1.29) |  |
| 40.1-126 | 1,031,565 | 487 | 0.97 (0.80-1.16) |  |
| *p* for trend |  |  | 0.620 |  |

HR, hazard ratios; CI, confidence interval; TC, total cholesterol; HDL-c, high-density lipoprotein cholesterol; LDL-c, low-density lipoprotein cholesterol; TC, total cholesterol, TG, triglycerides, HbA1c, haemoglobin A1c.

^1^ Adjusted for age (as the time scale of the model), sex, Townsend deprivation index, and use of anti-diabetic medications (as a time-varying covariate in the HbA1c cohort).

**eTable 23**. Association of the first measurement of creatinine, creatine kinase, and HbA1c with the incidence of frontotemporal dementia after excluding those with a history of ALS diagnosis. For each biomarker, the first row represents its association modelled continuously (per 1-SD increase), while subsequent rows evaluate the association categorically across tertiles, with the first tertile serving as the reference group.

| **Biomarker** | **N** | **No of events** | **Adjusted^1^**  **HR (95%CI)** | **P-value** |
| --- | --- | --- | --- | --- |
| **Creatinine (umol/L)** | 4,595,985 | 760 | 0.91 (0.84-0.98) | 0.003 |
| 45-71 | 1,451,749 | 152 | Reference |  |
| 71.1-86 | 1,538,503 | 256 | 1.01 (0.82-1.23) |  |
| 86.1-160 | 1,605,733 | 352 | 0.88 (0.71-1.10) |  |
| *p* for trend |  |  | 0.176 |  |
| **Creatinine kinase (log_2_ iu/L)** | 494,718 | 137 | 1.06 (0.90-1.25) | 0.463 |
| 25-76 | 159,701 | 41 | Reference |  |
| 76.6-122 | 166,957 | 42 | 0.94 (0.61-1.46) |  |
| 123-800 | 168,060 | 54 | 1.21 (0.78-1.88) |  |
| *p* for trend |  |  | 0.393 |  |
| **HbA1c (log_2_ mmol/L)** | 1,539,456 | 157 | 0.96 (0.85-1.09) | 0.528 |
| 27-36 | 465,991 | 36 | Reference |  |
| 36.1-40 | 572,994 | 51 | 0.53 (0.39-0.71) |  |
| 40.1-126 | 500,471 | 70 | 0.58 (0.44-0.76) |  |
| *p* for trend |  |  | 0.0004 |  |

HR, hazard ratios; CI, confidence interval; TC, total cholesterol; HDL-c, high-density lipoprotein cholesterol; LDL-c, low-density lipoprotein cholesterol; TC, total cholesterol, TG, triglycerides, HbA1c, haemoglobin A1c.

^1^ Adjusted for age (as the time scale of the model), sex, Townsend deprivation index, and use of anti-diabetic medications (as a time-varying covariate in the HbA1c cohort).

**eTable 24**. Association of the first measurement of creatinine, creatine kinase, and HbA1c with the incidence of amyotrophic lateral sclerosis adjusting for further covariates. For each biomarker, the first row represents its association modelled continuously (per 1-SD increase), while subsequent rows evaluate the association categorically across tertiles, with the first tertile serving as the reference group.

| **Biomarker** | **N** | **No of events** | **Adjusted^1^**  **HR (95%CI)** | **P-value** |
| --- | --- | --- | --- | --- |
| **Creatinine (umol/L)** | 4,598,895 | 2,687 | 0.96 (0.92-1.00) | 0.085 |
| 45-71 | 1,452,315 | 503 | Reference |  |
| 71.1-86 | 1,539,497 | 880 | 1.05 (0.94-1.17) |  |
| 86.1-160 | 1,607,083 | 1,304 | 1.01 (0.90-1.13) |  |
| *p* for trend |  |  | 0.916 |  |
| **Creatinine kinase (log_2_ iu/L)** | 495,195 | 437 | 1.12 (1.02-1.24) | 0.021 |
| 25-76 | 159,846 | 122 | Reference |  |
| 76.6-122 | 167,109 | 141 | 1.06 (0.83-1.36) |  |
| 123-800 | 168,240 | 174 | 1.30 (1.02-1.65) |  |
| *p* for trend |  |  | 0.028 |  |
| **HbA1c (log_2_ mmol/L)** | 2,888,132 | 984 | 0.98 (0.92-1.05) | 0.612 |
| 27-36 | 982,506 | 201 | Reference |  |
| 36.1-40 | 873,787 | 294 | 1.06 (0.89-1.27) |  |
| 40.1-126 | 1,031,839 | 489 | 0.91 (0.76-1.09) |  |
| *p* for trend |  |  | 0.221 |  |

HR, hazard ratios; CI, confidence interval; TC, total cholesterol; HDL-c, high-density lipoprotein cholesterol; LDL-c, low-density lipoprotein cholesterol; TC, total cholesterol, TG, triglycerides, HbA1c, haemoglobin A1c.

^1^ Adjusted for age (as the time scale of the model), sex, Townsend deprivation index, and use of anti-diabetic medications (as a time-varying covariate in the HbA1c cohort), smoking, BMI, comorbidities (cardiovascular diseases, peripheral vascular diseases, atrial fibrillation, diabetes, chronic kidney disease).

**eTable 25**. Association of the first measurement of creatinine, creatine kinase, and HbA1c with the incidence of frontotemporal dementia adjusting for further covariates. For each biomarker, the first row represents its association modelled continuously (per 1-SD increase), while subsequent rows evaluate the association categorically across tertiles, with the first tertile serving as the reference group.

| **Biomarker** | **N** | **No of events** | **Adjusted^1^**  **HR (95%CI)** | **P-value** |
| --- | --- | --- | --- | --- |
| **Creatinine (umol/L)** | 4,599,745 | 779 | 0.91 (0.84-0.99) | 0.022 |
| 45-71 | 1,452,572 | 161 | Reference |  |
| 71.1-86 | 1,539,731 | 259 | 0.96 (0.78-1.17) |  |
| 86.1-160 | 1,607,442 | 359 | 0.86 (0.69-1.05) |  |
| *p* for trend |  |  | 0.107 |  |
| **Creatinine kinase (log_2_ iu/L)** | 495,399 | 141 | 1.09 (0.92-1.30) | 0.330 |
| 25-76 | 159,876 | 41 | Reference |  |
| 76.6-122 | 167,166 | 45 | 1.03 (0.67-1.57) |  |
| 123-800 | 168,357 | 55 | 1.29 (0.85-1.97) |  |
| *p* for trend |  |  | 0.213 |  |
| **HbA1c (log_2_ mmol/L)** | 2,888,626 | 366 | 0.99 (0.88-1.10) | 0.788 |
| 27-36 | 982,642 | 105 | Reference |  |
| 36.1-40 | 873,962 | 83 | 0.55 (0.41-0.73) |  |
| 40.1-126 | 1,032,022 | 178 | 0.61 (0.47-0.79) |  |
| *p* for trend |  |  | 0.001 |  |

HR, hazard ratios; CI, confidence interval; TC, total cholesterol; HDL-c, high-density lipoprotein cholesterol; LDL-c, low-density lipoprotein cholesterol; TC, total cholesterol, TG, triglycerides, HbA1c, haemoglobin A1c.

^1^ Adjusted for age (as the time scale of the model), sex, Townsend deprivation index, and use of anti-diabetic medications (as a time-varying covariate in the HbA1c cohort), smoking, BMI, comorbidities (cardiovascular diseases, peripheral vascular diseases, atrial fibrillation, diabetes, chronic kidney disease).

**eTable 26**. Association (per 1-SD increase) of genetically predicted levels of creatinine and HbA1c with amyotrophic lateral sclerosis (ALS) and frontotemporal dementia (FTD) based on the two-sample Mendelian randomisation analysis.

| **Exposure** | **Outcome** | **MR Method** | **nSNP** | **OR** | **95%LCI** | **95%UCI** | **P-value** |
| --- | --- | --- | --- | --- | --- | --- | --- |
| Creatinine (Armstrong et al.) | ALS | Inverse variance weighted | 264 | 1.00 | 0.93 | 1.07 | 0.9971 |
| Creatinine (Armstrong et al.) | ALS | Weighted median | 264 | 1.08 | 0.97 | 1.20 | 0.1619 |
| Creatinine (Armstrong et al.) | ALS | MR Egger | 264 | 0.88 | 0.74 | 1.04 | 0.1284 |
| Creatinine (Armstrong et al.) | ALS | MR Egger intercept | 264 | - | - | - | 0.0920 |
| Creatinine (Armstrong et al.) | ALS | MR-RAPS | 264 | 0.99 | 0.93 | 1.05 | 0.6879 |
| Creatinine (Armstrong et al.) | ALS | MR PRESSO | 262 | 1.00 | 0.93 | 1.07 | 0.9267 |
| HbA1c (Wheeler et al.) | ALS | Inverse variance weighted | 36 | 0.96 | 0.71 | 1.30 | 0.7904 |
| HbA1c (Wheeler et al.) | ALS | Weighted median | 36 | 0.99 | 0.70 | 1.41 | 0.9575 |
| HbA1c (Wheeler et al.) | ALS | MR Egger | 36 | 0.97 | 0.55 | 1.73 | 0.9275 |
| HbA1c (Wheeler et al.) | ALS | MR Egger intercept | 36 | - | - | - | 0.9546 |
| HbA1c (Wheeler et al.) | ALS | MR-RAPS | 36 | 0.96 | 0.76 | 1.21 | 0.7240 |
| HbA1c (Wheeler et al.) | ALS | MR PRESSO | 35 | 1.00 | 0.76 | 1.31 | 0.9794 |
| Creatinine (Armstrong et al.) | FTD | Inverse variance weighted | 249 | 0.73 | 0.56 | 0.96 | 0.0219 |
| Creatinine (Armstrong et al.) | FTD | Weighted median | 249 | 0.81 | 0.54 | 1.21 | 0.2971 |
| Creatinine (Armstrong et al.) | FTD | MR Egger | 249 | 0.68 | 0.37 | 1.25 | 0.2153 |
| Creatinine (Armstrong et al.) | FTD | MR Egger intercept | 249 | - | - | - | 0.7806 |
| Creatinine (Armstrong et al.) | FTD | MR-RAPS | 249 | 0.77 | 0.54 | 1.00 | 0.0272 |
| Creatinine (Armstrong et al.) | FTD | MR PRESSO | 247 | 0.79 | 0.61 | 0.99 | 0.0489 |
| HbA1c (Wheeler et al.) | FTD | Inverse variance weighted | 31 | 1.01 | 0.38 | 2.68 | 0.9855 |
| HbA1c (Wheeler et al.) | FTD | Weighted median | 31 | 0.85 | 0.19 | 3.88 | 0.8376 |
| HbA1c (Wheeler et al.) | FTD | MR Egger | 31 | 4.43 | 0.67 | 29.10 | 0.1326 |
| HbA1c (Wheeler et al.) | FTD | MR Egger intercept | 31 | - | - | - | 0.0860 |
| HbA1c (Wheeler et al.) | FTD | MR-RAPS | 31 | 1.11 | 0.43 | 2.84 | 0.8355 |

## References

1. The 1000 Genomes Project Consortium, Corresponding authors, Auton A, Abecasis GR, Steering committee, Altshuler DM, et al. A global reference for human genetic variation. Nature. 2015 Oct 1;526(7571):68–74.

2. Sinnott-Armstrong N, Tanigawa Y, Amar D, Mars N, Benner C, Aguirre M, et al. Genetics of 35 blood and urine biomarkers in the UK Biobank. Nat Genet. 2021 Feb;53(2):185–94.

3. Wheeler E, Leong A, Liu CT, Hivert MF, Strawbridge RJ, Podmore C, et al. Impact of common genetic determinants of Hemoglobin A1c on type 2 diabetes risk and diagnosis in ancestrally diverse populations: A transethnic genome-wide meta-analysis. Gregg E, editor. PLOS Med. 2017 Sep 12;14(9):e1002383.

4. Hemani G, Zheng J, Elsworth B, Wade KH, Haberland V, Baird D, et al. The MR-Base platform supports systematic causal inference across the human phenome. eLife. 2018 May 30;7:e34408.

5. Lawlor DA, Harbord RM, Sterne JAC, Timpson N, Davey Smith G. Mendelian randomization: Using genes as instruments for making causal inferences in epidemiology. Stat Med. 2008 Apr 15;27(8):1133–63.

6. Van Rheenen W, Van Der Spek RAA, Bakker MK, Van Vugt JJFA, Hop PJ, Zwamborn RAJ, et al. Common and rare variant association analyses in amyotrophic lateral sclerosis identify 15 risk loci with distinct genetic architectures and neuron-specific biology. Nat Genet. 2021 Dec;53(12):1636–48.

7. Brooks BR, Miller RG, Swash M, Munsat TL. El Escorial revisited: Revised criteria for the diagnosis of amyotrophic lateral sclerosis. Amyotroph Lateral Scler Other Motor Neuron Disord. 2000 Jan;1(5):293–9.

8. Ferrari R, Hernandez DG, Nalls MA, Rohrer JD, Ramasamy A, Kwok JBJ, et al. Frontotemporal dementia and its subtypes: a genome-wide association study. Lancet Neurol. 2014 Jul;13(7):686–99.

9. Faber R, Neary D. Frontotemporal lobar degeneration: A consensus on clinical diagnostic criteria. Neurology. 1999 Sep 1;53(5):1158–1158.

10. Bowden J, Del Greco M F, Minelli C, Davey Smith G, Sheehan N, Thompson J. A framework for the investigation of pleiotropy in two‐sample summary data Mendelian randomization. Stat Med. 2017 May 20;36(11):1783–802.

11. Bowden J, Davey Smith G, Burgess S. Mendelian randomization with invalid instruments: effect estimation and bias detection through Egger regression. Int J Epidemiol. 2015 Apr 1;44(2):512–25.

12. Bowden J, Davey Smith G, Haycock PC, Burgess S. Consistent Estimation in Mendelian Randomization with Some Invalid Instruments Using a Weighted Median Estimator. Genet Epidemiol. 2016 May;40(4):304–14.

13. Hartwig FP, Davey Smith G, Bowden J. Robust inference in summary data Mendelian randomization via the zero modal pleiotropy assumption. Int J Epidemiol. 2017 Dec 1;46(6):1985–98.

14. Verbanck M, Chen CY, Neale B, Do R. Detection of widespread horizontal pleiotropy in causal relationships inferred from Mendelian randomization between complex traits and diseases. Nat Genet. 2018 May;50(5):693–8.

15. Zhao Q, Wang J, Hemani G, Bowden J, Small DS. Statistical inference in two-sample summary-data Mendelian randomization using robust adjusted profile score. Ann Stat. 2020;48(3):1742–69.
